# Glioma Image-Level and Slide-Level Gene Predictor (GLISP) for Molecular Diagnosis and Predicting Genetic Events of Adult Diffuse Glioma

**DOI:** 10.3390/bioengineering12010012

**Published:** 2024-12-27

**Authors:** Minh-Khang Le, Masataka Kawai, Kenta Masui, Takashi Komori, Takakazu Kawamata, Yoshihiro Muragaki, Tomohiro Inoue, Ippei Tahara, Kazunari Kasai, Tetsuo Kondo

**Affiliations:** 1 Department of Pathology, University of Yamanashi, Yamanashi 409-3898, Japan; minhkhangle.phd@gmail.com (M.-K.L.); tomohiroi@yamanashi.ac.jp (T.I.); itahara@yamanashi.ac.jp (I.T.); kasaik@yamanashi.ac.jp (K.K.); ktetsuo@yamanashi.ac.jp (T.K.); 2 Department of Pathology, Tokyo Women’s Medical University, Tokyo 162-8666, Japan; masui-kn@twmu.ac.jp; 3 Department of Laboratory Medicine and Pathology (Neuropathology), Tokyo Metropolitan Neurological Hospital, Tokyo 183-0042, Japan; komori-tk@igakuken.or.jp; 4 Department of Neurosurgery, Tokyo Women’s Medical University, Shinjuku, Tokyo 162-8666, Japan; tkawamata@twmu.ac.jp; 5 Center for Advanced Medical Engineering Research and Development, Kobe University, Kobe 650-0047, Hyogo, Japan; ymuragaki@gmail.com

**Keywords:** glioma, artificial intelligence, deep learning, new WHO classification, genetic abnormalities

## Abstract

The latest World Health Organization (WHO) classification of central nervous system tumors (WHO2021/5th) has incorporated molecular information into the diagnosis of each brain tumor type including diffuse glioma. Therefore, an artificial intelligence (AI) framework for learning histological patterns and predicting important genetic events would be useful for future studies and applications. Using the concept of multiple-instance learning, we developed an AI framework named GLioma Image-level and Slide-level gene Predictor (GLISP) to predict nine genetic abnormalities in hematoxylin and eosin sections: *IDH1/2*, *ATRX*, *TP53* mutations, *TERT* promoter mutations, *CDKN2A/B* homozygous deletion (CHD), *EGFR* amplification (*EGFR*amp), 7 gain/10 loss (7+/10−), 1p/19q co-deletion, and *MGMT* promoter methylation. GLISP consists of a pair of patch-level GLISP-P and patient-level GLISP-W models, each pair of which is for a genetic prediction task, providing flexibility in clinical utility. In this study, the Cancer Genome Atlas whole-slide images (WSIs) were used to train the model. A total of 108 WSIs from the Tokyo Women’s Medical University were used as the external dataset. In cross-validation, GLISP yielded patch-level/case-level predictions with top performances in *IDH1/2* and 1p/19q co-deletion with average areas under the curve (AUCs) of receiver operating characteristics of 0.75/0.79 and 0.73/0.80, respectively. In external validation, the patch-level/case-level AUCs of *IDH1/2* and 1p/19q co-deletion detection were 0.76/0.83 and 0.78/0.88, respectively. The accuracy in diagnosing IDH-mutant astrocytoma, oligodendroglioma, and IDH-wild-type glioblastoma was 0.66, surpassing the human pathologist average of 0.62 (0.54–0.67). In conclusion, GLISP is a two-stage AI framework for histology-based prediction of genetic events in adult gliomas, which is helpful in providing essential information for WHO 2021 molecular diagnoses.

## 1. Introduction

Brain tumors were traditionally classified based solely on their morphological features. Over the past decade, brain tumor research has repeatedly shown that molecular features reflect the biological behavior of brain tumors more consistently and accurately than histological features assessed by pathologists [1]. The World Health Organization (WHO) adopted a molecular classification for adult diffuse gliomas in the updated 4th edition of the Classification of Tumors of the Central Nervous System (CNS) in 2016 for the first time [2], making a major advancement from the 3rd edition (WHO 2007). An updated 2021 WHO Classification of Tumors of CNS 5th edition (WHO 2021) further advanced molecular classification into the grading of adult and pediatric-type diffuse gliomas [3,4,5]. Consequently, traditional histology-based diagnostics have been replaced by a multi-layered approach that combines histological features and molecular information in an integrated manner. However, the incorporation of molecular genetic information into the “routine diagnostics” of each brain tumor type to achieve precision medicine in the clinic remains a major challenge. This is rendered more challenging in low- and middle-income countries because of high costs, lack of technological infrastructure, lack of skilled technical human resources, and a shortage of trained neuropathologists [6].

In the context of practical artificial intelligence (AI) algorithms applied to investigate brain tumors, numerous models have been devised to prognose genetic occurrences. However, AI research that considers the new diagnostic workflow of the 5th edition of the WHO guidelines is notably scarce. Furthermore, AI investigations related to brain tumors are primarily concentrated in the field of medical imaging. Therefore, it is essential to develop machine learning-based AI models using hematoxylin and eosin (H&E) sections to predict the genotypes of brain tumors for better classification according to the updated WHO 2021.

Adult-type diffuse gliomas are prevalent CNS tumors and include three genotype-based, peculiar types of tumors: (1) astrocytoma, IDH-mutant, (2) oligodendroglioma, IDH-mutant and 1p/19q-co-deleted, and (3) glioblastoma (GBM), IDH-wildtype [7]. Astrocytoma and oligodendroglioma share the cardinal mutation of isocitrate dehydrogenase (IDH) genes while only oligodendroglioma is associated with the co-deletion of chromosome arms 1p and 19q. GBM is characterized by wild-type IDH genes with the so-called GBM genotypes, such as *TERT* promoter mutation, *EGFR* gene amplification, and gain of chromosome 7 plus loss of chromosome 10 (7+/10−). Notably, molecular findings can also be used to define the malignancy (grading) of brain tumors. For IDH-mutant astrocytomas, the acquisition of *CDKN2A/B* homozygous deletion corresponds to CNS WHO grade 4, even in the absence of high-grade histological findings (necrosis and microvascular proliferation [MVP]) [3]. Furthermore, *MGMT* methylation is a predictive biomarker of response to temozolomide in high-grade gliomas. Adult-type diffuse gliomas are theoretically ideal neoplasms for designing AI algorithms to predict molecular findings and refine genotype-based classifications. These methods provide an opportunity to ease the budget burden of molecular tests [8]. Immunohistochemistry for IDH1 R132H protein and fluorescence in situ hybridization (FISH) analysis for 1p/19q co-deletion has become more feasible and popular. However, other genetic events are still difficult to determine in routine diagnosis.

Therefore, the authors of the present study developed an AI model utilizing deep convolutional neural networks (CNN) and an attention-based meta-model, designed to extract the molecular genetic characteristics of adult-type diffuse glioma, which are crucial for diagnosis according to the 5th edition of the WHO guidelines. The model was trained using two extensive histopathological datasets from the Cancer Genome Atlas (TCGA). We named this AI framework the GLioma Image-level and Slide-level gene Predictor (GLISP), which is designed to predict the key genetic aberrations of gliomas. The emphasis is on the application of this model in line with the 5th edition of the WHO guidelines. GLISP works as a flexible diagnostic tool that complements human-based morphological findings with genetic insights and fills incomplete pieces for molecular diagnosis. Our model put a further, but not perfect, step in the initial histopathological diagnosis of adult-type diffuse glioma, particularly in low to middle-income institutions where molecular testing is limited.

## 2. Method and Materials

### 2.1. Data Preparation and Patch Preprocessing

**Figure 1:**
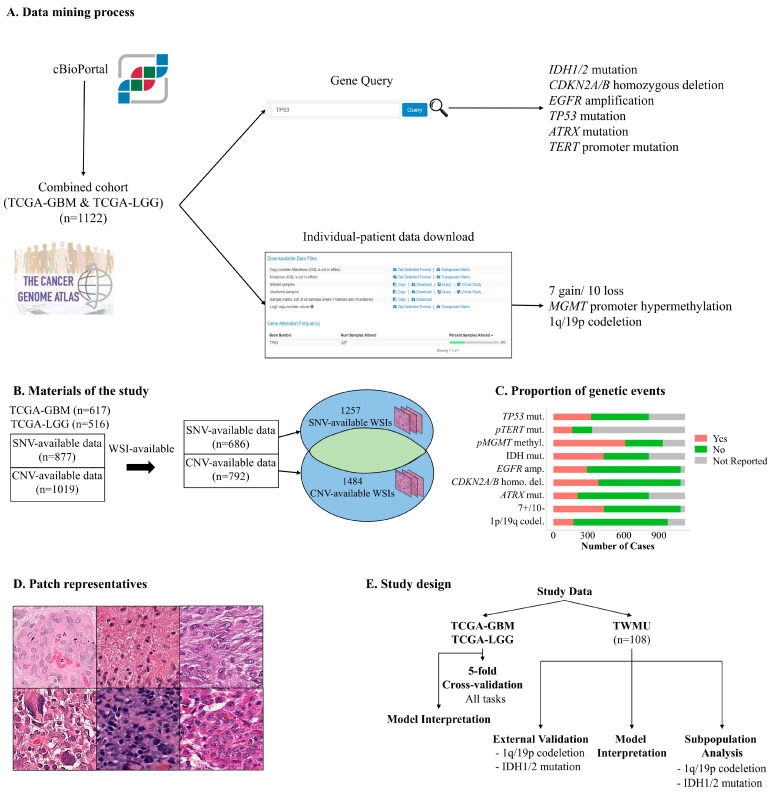
(**A**) Data mining process. We accessed cBioportal to retrieve the information of filtered and high-quality information about genetic events, including *IDH1/2*, *ATRX*, *TP53*, and *TERT* promoter mutations, *CDKN2A/B* homozygous deletion, *EGFR* amplification, 7 gain/10 loss chromosomal abnormalities, 1p/19q co-deletion, and *MGMT* hypermethylation. (**B**) Materials of the study. Among 1123 cases (n = 617 of the TCGA-GBM and n = 516 of the TCGA-LGG), simple nucleotide variation (SNV) and copy number variation (CNV) are available in 877 and 1019 cases, respectively. The number of corresponding SNV-available and CNV-available slides are 1257 and 1484, respectively. (**C**) The bar graph shows the availability of data in the 9 genetic events of interest. (**D**) The representatives of 256 × 256 pixels are used in the deep learning training process. (**E**) The study design. Since the data is heterogeneous, the cases are randomly assigned to train and test data, independently of other tasks. TCGA: The Cancer Genome Atlas. WSI: whole slide image. TWMU: Tokyo Women’s Medical University.

Figure 1 illustrates the data preparation process. TCGA contains two glioma-related datasets: the TCGA-GBM (617 patients) and TCGA-LGG (516 patients). Case-level information on simple nucleotide variations (SNV), copy number variations (CNV), and other clinical parameters were retrieved. From the two datasets, we extracted 877 SNV-available and 1019 CNV-available patients. SNV and CNV information was extracted from cBioPortal (Figure 1A) [9,10,11], which is an open-access, open-source web resource for interactive exploration and analysis of multidimensional cancer genomics data sets, including TCGA datasets. Information on *IDH1/2*, *ATRX*, *TP53* mutations, *TERT* promoter mutations, *EGFR* amplification (*EGFR*amp), *CDKN2A/B* homozygous deletion (CHD), 7 gain 10 loss chromosomal aberrations, 1p/19q co-deletion, and *MGMT* promoter methylation was collected from this portal.

There was a total of 846 and 859 whole slide image (WSI) files in the TCGA-LGG and TCGA-GBM projects, respectively. We revisited all WSIs and removed repeated, low-qualified, immunohistochemical, and tumor-paucity slides, leaving 1584 H&E-stained WSIs. A pathologist (M.-K.L.) annotated the regions of interest (ROIs) where tumor cells were located in each WSI. Finally, there were 686 SNV-available and 792 CNV-available cases, with 1257 SNV-available and 1484 CNV-available WSI files, from which we generated approximately 50 patches per file (Figure 1B). Patches with more than 50% whitespace (determined by simply thresholding the color channels) were eliminated and subsequently revised again by the same pathologist. There were 67,760 SNV-available and 77,942 CNV-available patches with a resolution of 256 × 256 pixels. Figure 1C shows the data available for genetic events. The actual size of the patches is 125 µm × 125 µm (0.5 microns per pixel), which can capture important pathological findings such as MVP or palisading necrosis (Figure 1D).

As the data availability of genetic events was not homogenous, we performed a 5-fold cross-validation for each task. Therefore, the training and validation cases of a task were different from those of other tasks in cross-validation (Figure 1E). For external validation, we collected 108 WSIs from Tokyo Women’s Medical University (TWMU), including 38 IDH-mutant astrocytomas, 40 oligodendrogliomas with IDH mutation and 1p/19q co-deletion, and 30 IDH-wildtype glioblastomas. IDH mutation and 1p/19q co-deletion were identified using multiplex ligation-dependent probe amplification, as previously reported. The patient information for this dataset (n = 108) is reported in Table 1. The patch-level and WSI-level predictions of GLISP on this dataset were evaluated.

### 2.2. GLISP Framework and Training Process

**Figure 2:**
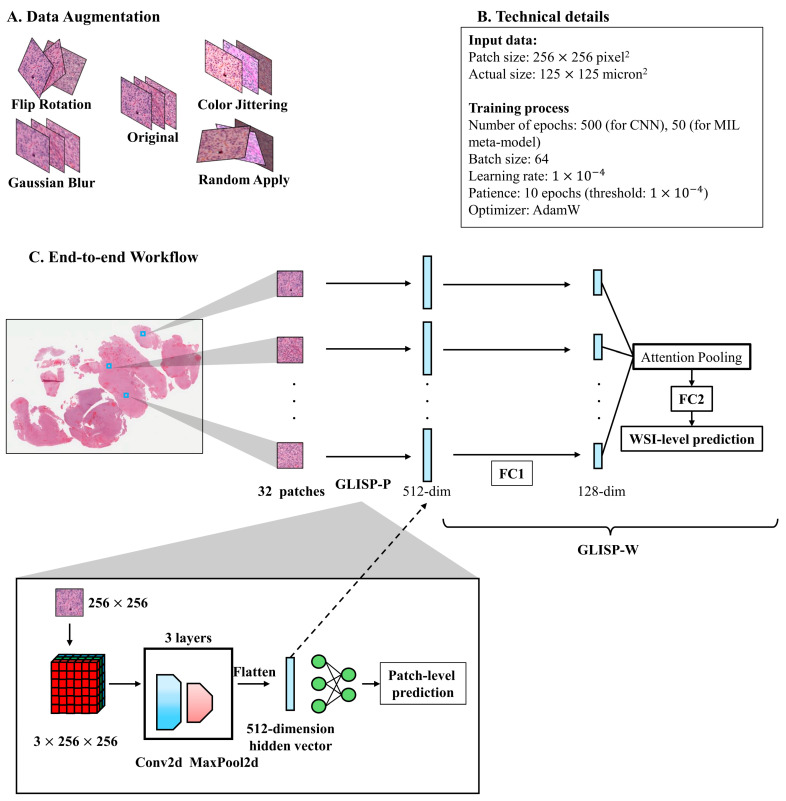
(**A**) In the training process, random flipping, random rotation, color jittering, and Gaussian blurring of the training patches are randomly applied for data augmentation. (**B**) The table shows the technical details of input data and hyperparameters of the training process. (**C**) The architecture of the patch-level model (GLISP-P) includes four layers of convolutional operations and a fully connected layer. The structure of the GLISP-W consists of two multi-layer perceptrons (MLP) with an attention-pooling operation in between. MIL: multiple instance learning. FC: fully connected layer.

During the training process, the patches underwent image data augmentation, including flip/rotation, color jittering, Gaussian blurring, and randomly applied mixed distortion of the above augmentations with a probability of 0.5 (Figure 2A). Since we found color normalization unnecessary in our training context, no color normalization was included in the augmentation protocol. The technical details are shown in Figure 2B. First, we created a deep learning model, which is named GLISP-P, to predict patch-level prediction. To decide on the GLISP-P architecture, we carried out experiments within the IDH cohort, which comprised 20 training cases and 10 validation cases, ensuring an even split between mutant and wild-type instances. We trained a range of models: a medium-sized vision transformer, Inception V3, ShuffleNetv2, and a small customed CNN. The first three models were pre-trained on ImageNet, while the small CNN started from a random initialization. Their validation accuracies were 0.68, 0.63, 0.71, and 0.67, in that order. The comparison between the top-performing ShuffleNetv2 and the small CNN yielded a *p*-value of 0.885. Given its efficiency in computation, we chose the compact CNN architecture for GLISP-P. The customed CNN is a small CNN with four convolutional layers followed by a multi-layer perceptron (MLP). The output is the probability of each genetic event (Figure 2C).

The convolutional operation layer t can be expressed by the following formula:

$$X^{t+1}(c)=b^{c}+\sum_{k=0}^{C^{t}-1}W\left(c,k\right)\times X^{t}(k)$$

$X^{t}$, $X^{t+1}$ are the input $\left(C^{t},H^{t},W^{t}\right)$ and output $\left(C^{t+1},H^{t+1},W^{t+1}\right)$ feature maps of the convolutional layer *t* while *c* represents each channel of $C^{t+1}$ and $b^{c}$ is the bias term of the channel c.

The linear layer t of the MLP module can be expressed by the following formula:

$$X^{t+1}=X^{t}W^{t}+b$$

$X^{t},X^{t+1}$ are the input and output features with dimensions $C^{t}$ and $C^{t+1}$ while $W^{t}$ is a $\left(C^{t},C^{t+1}\right)$ weight matrix. *b* is the bias term for the current output channel $C^{t+1}$.

For WSI-level prediction, we trained GLISP-W, a neural network meta-model with two MLP layers (MLP1 and MLP2) and an attention-pooling layer in between them. This can be considered as a type of multiple-instance learning (MIL) 12. The GLISP-P and GLISP-W models constitute the GLISP framework. Full descriptions of GLISP-P and GLISP-W are depicted in Supplementary File Part 1, Figure S1.

### 2.3. Human Evaluation

Three Japanese board-certified pathologists, each with nine to eleven years of experience, were recruited to evaluate TWMU’s external evaluation data. Although they are not specialized in neuropathology, they diagnose approximately 10 glioma cases per year at the University of Yamanashi Hospital. They blindly and independently classified each WSI into one of the diagnoses, including astrocytoma, oligodendroglioma, and glioblastoma, according to WHO 2021, based solely on H&E sections. This evaluation aimed to create a pathologist-level performance baseline and examine whether the model can outperform this baseline. A better molecular classification compared to human diagnosis can be helpful in the diagnostic workflow of adult glioma.

### 2.4. Human-AI Integrative Diagnostic Workflow

**Figure 3:**
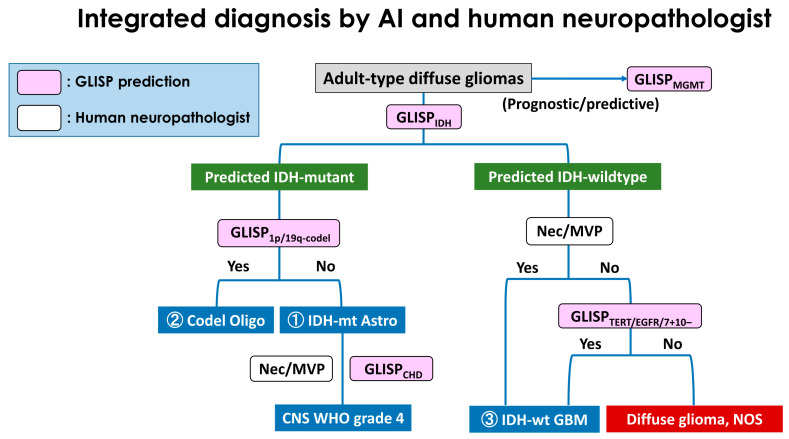
The GLISP-incorporating diagnostic workflow performs AI-based molecular diagnosis. GLISP models are beneficial in an integrated workflow. First, the pathologist examines and recognizes the glioma morphology of a brain tumor. GLISP_IDH_ then categorizes the tumor based on IDH status. If an IDH-mutant phenotype is predicted, subsequent examinations of molecular statuses by GLISP_1p/19q-codel_, GLISP_CHD_, and the presence of microvascular proliferation (MVP) and/or necrosis can provide a more molecular-integrated diagnosis compared to the classic diagnosis. If an IDH-wildtype phenotype is detected, MVP/necrosis, as well as molecular statuses predicted by GLISP_TERT_, GLISP_EGFR_, and GLISP_7+10−_, can be useful for further molecular diagnosis.

The GLISP-incorporating molecular diagnoses were made using the WHO 2021 diagnostic workflow (Figure 3). First, the pathologist examines and recognizes the glioma morphology of a brain tumor. GLISP_IDH_ then categorizes the tumor based on IDH status. If an IDH-mutant phenotype is predicted, subsequent examinations of molecular statuses by GLISP_1p/19q-codel_, GLISP_CHD_, and the presence of microvascular proliferation (MVP) and/or necrosis can provide a more molecular-integrated diagnosis compared to the classic diagnosis. If an IDH-wildtype phenotype is detected, MVP/necrosis, as well as molecular statuses predicted by GLISP_TERT_, GLISP_EGFR_, and GLISP_7+10−_, can be useful for further molecular diagnosis.

### 2.5. Subpopulation Analyses

Given the context of clinical utility, we evaluated GLISP in certain practical contexts: (1) given IDH1/2-wildtype phenotype, GLISP predicts TERT promoter mutation, EGFRamp, and 7+/10− for glioblastoma diagnosis; (2) given the IDH-mutant phenotype, GLISP predicts CHD for astrocytoma, grade 4 diagnosis; and (3) given the IDH-mutant phenotype, GLISP predicts 1p/19q co-deletion for oligodendroglioma diagnosis. These contexts emphasize the specific key points in human-AI integrative workflow (Figure 3), where AI can be helpful. Evaluating AI in these specific contexts is more pathologically practical than measuring its performance in a general context.

### 2.6. Analysis Platform

All procedures were performed using Python version 3.8 (The Python Software Foundation, Wilmington, DE, USA). To create histopathological patches, we used the OpenSlide 0.9.1 package. We constructed the deep-learning models and conducted the training process using Pytorch 1.8.1 and Torchvision 0.12.0 packages. Our local machine has 32 GB of RAM, an NVIDIA Geforce RTX 3080 Ti GPU, and a 16-core CPU with a 4.5 GHz clock rate run by 24 threads. The posthoc analyses as well as visualization were performed using the combination of Python 3.8 and R version 4.2.2 (The R Foundation, Vienna, Austria).

## 3. Results

### 3.1. Cross-Validation Results

The cross-validation results are presented in Table 2. The task with the highest performance was *IDH1/2* mutation prediction, which had patch-level and case-level receiver operating characteristics (ROC) area under the curve (AUC) of 0.75 (±0.028) and 0.79 (±0.039), respectively. The patch-level/case-level ROC AUC of *ATRX*, *TP53*, *TERT* promoter mutations, *EGFR*amp, CHD, 7+/10−, 1p/19q co-deletion, and *MGMT* promoter methylation prediction were 0.63 (±0.090)/0.65 (±0.109), 0.70 (±0.034)/0.70 (±0.029), 0.67 (±0.079)/0.73 (±0.083), 0.73 (±0.030)/0.78 (±0.029), 0.69 (±0.026)/0.74 (±0.032), 0.75 (±0.020)/0.78 (±0.019), 0.73 (±0.039)/0.80 (±0.052), and 0.64 (±0.052)/0.67 (±0.074), respectively. Supplementary File (Part 2, Figures S2–S10) demonstrates the individual ROC analysis of each fold in each genetic prediction task.

### 3.2. External Validation

**Figure 4:**
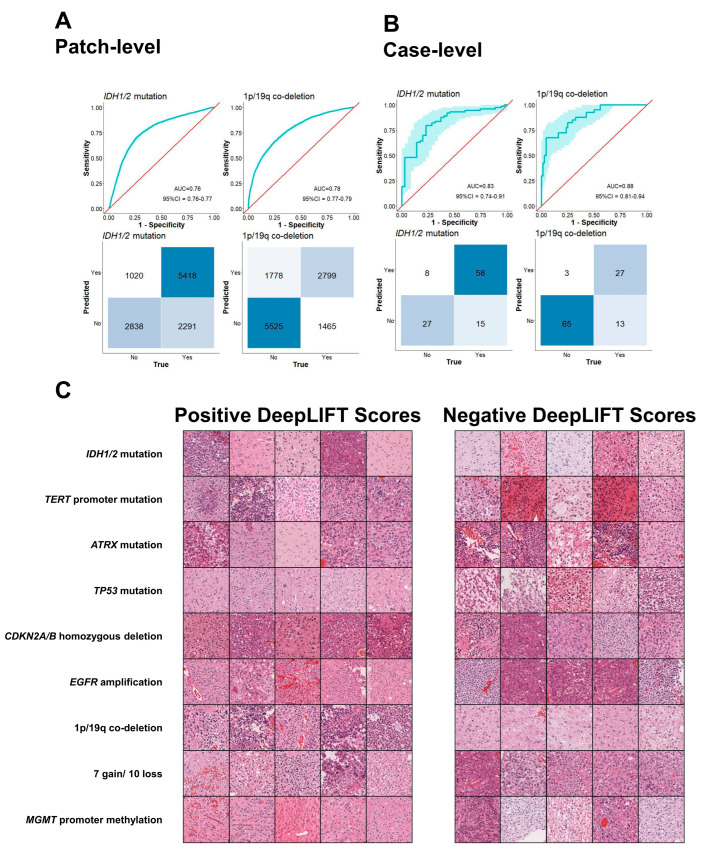
Patch-level (**A**) and WSI-level (**B**) evaluation of GLISP in predicting *IDH1/2* mutation and 1p/19q, including receiver operating characteristics (ROC) analysis and confusion matrix. (**C**) Most informative patches for positive (**left**) and negative (**right**) prediction of GLISP are measured by the patch-level DeepLIFT scores.

To validate the robustness of GLISP, we performed model testing on 108 slides from the TWMU. In this section, we picked GLISP models trained on fold 1 of each prediction task in the cross-validation process. Figure 4A,B show the results of the external validation. Only *IDH1/2* mutation and 1q/19p co-deletion were available labels in this dataset. The model achieved the patch-level and case-level ROC AUC of 0.76 (95%CI = 0.76–0.77)/0.83 (95%CI = 0.74–0.91) and 0.78 (95%CI = 0.77–0.79)/0.88 (95%CI = 0.81–0.94) in predicting *IDH1/2* mutation and 1q/19p co-deletion, respectively. Table 3 shows a confusion matrix comparing the GLISP-predicted and ground-truth molecular diagnoses, including IDH-mutant astrocytomas, oligodendrogliomas with IDH mutation and 1p/19q co-deletion, and IDH-wildtype glioblastomas. The overall accuracy was 0.66 (95%CI = 0.56–0.74) and F1 scores for each tumor class were 0.70 (95%CI = 0.58–0.80), 0.62 (95%CI = 0.46–0.76), and 0.64 (95%CI = 0.49–0.76), respectively. The accuracy exceeded the board-certified pathologists (non-neuropathologists) blinded evaluation average of 0.62 (95%CI = 0.57–0.74). The F1 scores for each tumor class all exceeded 0.6, showing consistent performances among classes compared to human baselines 0.57 (95%CI = 0.43–0.68), 0.74 (95%CI = 0.63–0.84), and 0.64 (95%CI = 0.45–0.78) (T.I.), 0.51 (95%CI = 0.33–0.65), 0.48 (95%CI = 0.32–0.63), and 0.59 (95%CI = 0.45–0.70) (I.T.), 0.63 (95%CI = 0.52–0.75), 0.52 (95%CI = 0.34–0.67), and 0.82 (95%CI = 0.71–0.91) (K.K.). The detailed results were reported in Supplementary File (Part 3).

### 3.3. Model Interpretation

To further understand how the model predicts genetic events, we employed the Deep Learning Important FeaTures (DeepLIFT) method [12] to trace the “negative” and “positive” morphological features, which are associated with the absence or presence of the genetic event. The output of this method for each patch is the tensor of the pixel-level scores. We calculated a patch-level score by averaging all pixel-level scores of the corresponding DeepLIFT output. Figure 4C illustrates the most representative patches with negative and positive patch-level DeepLIFT scores for all tasks. The morphological differences between “negative” and “positive” patches were not straightforward for human eyes. We also illustrated the pixel-level model interpretability, which seems to be challenging for interpreting the morphological features (Supplementary File, Part 4, Figures S11–S19).

### 3.4. Subpopulation Analyses

**Figure 5:**
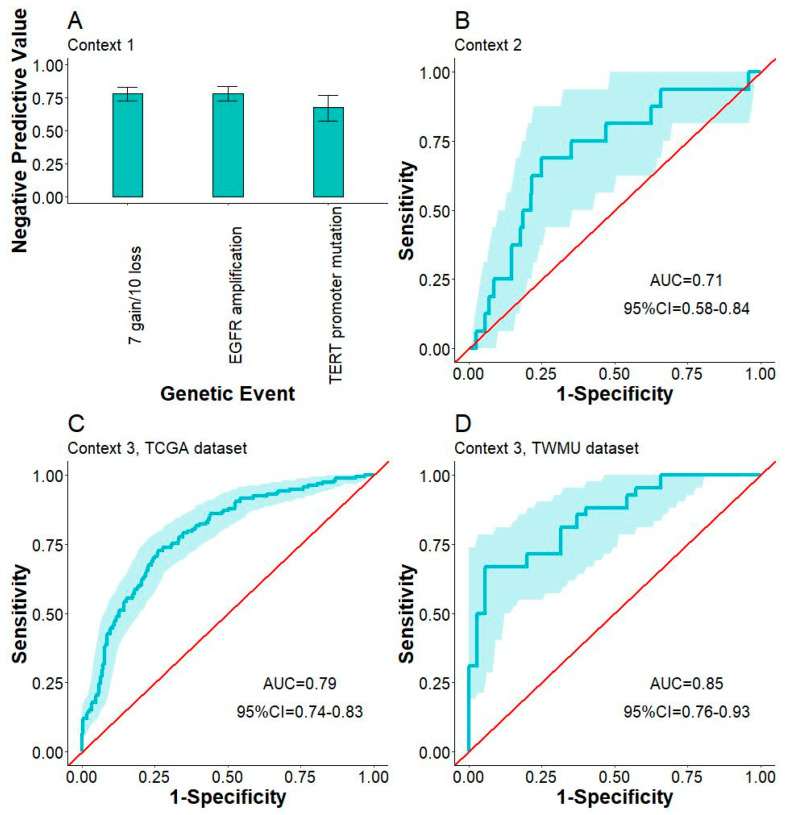
Subpopulation analysis. (**A**) The negative predictive values of predicting 7+/10–, *EGFR* amplification, *TERT* promoter mutation in IDH-wildtype phenotype (context 1). (**B**) The receiver operating characteristics (ROC) analysis with 95%CI band of predicting *CDKN2A/B* homozygous deletion in IDH-mutant phenotype (context 2). The ROC analyses of predicting 1p/19q co-deletion in IDH-mutant phenotype in TCGA (**C**) and TWMU datasets (**D**) (context 3).

Figure 5 summarizes the results of subpopulation analyses. In context 1, we found no IDH-wild-type glioma with ground-truth positivity for the three genetic events in the TCGA datasets. Therefore, we measured the performance of GLISP using negative predictive value (NPV). The NPVs of predicting *TERT* promoter mutation, *EGFR*amp, and 7+/10− were 0.68, 0.78, and 0.78, respectively. In context 2, AUC and accuracy in predicting CHD were 0.71 and 0.75, respectively, while those metrics in predicting 1p/19q co-deletion in context 3 were 0.79 and 0.73, respectively. We also performed external validation of Context 3. The AUC and accuracy in predicting 1p/19q co-deletion in context 3 were 0.85 and 0.79, respectively. In IDH-wildtype glioblastoma, the ROC AUC of detecting MGMT promoter hypermethylation of GLISP was 0.58 (95%CI = 0.49–0.66).

## 4. Discussion

### 4.1. Principal Findings

Our study illustrates the potential associations between morphology and the underlying genetic event in adult diffuse gliomas. By predicting the genetic status based on histological patterns, pathologists can accelerate the diagnostic workup of CNS neoplasms. Clinically, it is helpful for tumor classification because the new WHO 2021 terminology requires genetic testing. This requirement can be challenging for some institutions, especially for those in developing countries. There are several diagnostic scenarios where genetic testing or prediction is a crucial factor in the decision-making process. Given glioma histology, GLISP prediction of *IDH1/2* mutation is diagnostically and prognostically important. In the IDH-mutant glioma, predicting 1p/19q co-deletion and CDH is pivotal in classifying and grading the tumors. Meanwhile, detecting *EGFR*amp, p*TERT*, and 7+/ 10– is valuable in grading IDH-wildtype glioma. This study presents several points for discussion. First, based on our results, genetic abnormalities were related to histopathological patterns via model computation, although this relationship is not straightforward in the human sense. Second, GLISP can be integrated into the molecular diagnostic workflow without conflicting with human pathologists. Human pathologists are not trained to differentiate genetic events and instead rely on genetic testing, though they reliably identify MVP and necrosis. In this sense, GLISP and human pathologists complement each other rather than compete. Third, the GLISP blocks in the workflow (Figure 3) are arranged flexibly according to the availability of genetic testing. For example, when FISH analysis for 1p/19q co-deletion is available, GLISP_1p/19q_ can be substituted. In another scenario, immunohistochemistry for *IDH1 R132H* can replace GLISP_IDH_ in older patients. While an end-to-end model trained to target the final molecular diagnosis might perform better, it lacks this flexibility.

Genetic analyses have become more important with the current WHO 2021 classification of CNS tumors. Gene mutations can alter tumor grading and treatment course [7]. A model that can utilize pathological data to predict genetic events is valuable because molecular information is not always obtainable, particularly in developing countries [13]. As previously discussed, different genetic events under the presence or absence of IDH status play important roles in the molecular classification of adult glioma. Given the complexity of the diagnostic context, investigating the molecular diagnostic capacity of GLISP is necessary to examine how the model functions in the real-life diagnostic flow. Our results showed a relatively more balanced and consistent performance of GLISP than morphological diagnosis by human pathologists.

In human evaluation, our results showed a relatively more balanced and consistent performance of GLISP than morphological diagnosis by human pathologists. Our analyses indicate that glioma diagnosis based on IDH mutation and 1p/19 co-deletion is challenging for human pathologists. Additionally, we focused on evaluating GLISP performances in specific contexts in which AI can aid values in the diagnostic workflow according to the recent WHO classification. GLISP showed balanced performance over classes and surpassed overall accuracy compared with morphological diagnosis baselines. We did not conduct similar external validation for *ATRX*, *TP53* mutations, and *MGMT* promoter methylation due to their lower diagnostic importance. Additionally, TERT promoter mutations, *EGFR*amp, CHD, and 7 gain 10 loss chromosomal aberrations were not validated because of the rarity of each event. Moreover, the human pathologists are less familiar with the morphological patterns associated with these genetic events compared to IDH mutations and 1p/19q co-deletion, resulting in unreliable baselines. GLISP performance to detect *MGMT* promoter hypermethylation was relatively low in comparison with other genetic events, which may come from the coincidence with IDH mutation. Further training on the IDH-wildtype cohort is an option to improve the performance.

### 4.2. Comparison to Related Works

Deep learning techniques have been developed in recent years, which have motivated research in medical domains dealing with computer vision-related problems and high-dimensional data such as imaging and pathology [14,15]. Histopathology-based prediction of recurrent mutations in diffuse adult glioma is challenging because of histologic variations and overlapping features between mutant and wild-type tumors for each of the important genes. A previous small study on 266 cases, including training, validation, and testing cohorts illustrated a deep-learning model predicting IDH mutations with the highest AUC of 0.93 [16]. Recently, an end-to-end deep-learning model integrating patch-based classification and majority voting was developed on a large cohort to predict the neuropathologist-level integrated classification of adult-type diffuse gliomas [17], and F1 scores for IDH-mutant astrocytoma, oligodendroglioma with IDH mutation and 1p/19q co-deletion, and IDH-wildtype glioblastomas were 0.81–0.87, 0.77–0.88, and 0.93–0.97, respectively. A few machine learning studies have attempted to perform such tasks, most of which are based on imaging features [18,19].

Other branches of glioma machine learning research have focused on gene expression patterns [20,21] and survival analyses [22]. In addition to predicting mutational status for diagnostic purposes, deep learning exploration of epigenetic patterns such as DNA methylation and chromatin modifications in brain tumors is also a potential approach [23,24]. Imaging-based prediction of adult glioma gene abnormalities has also been investigated [20]. However, an illustration of the robustness of these models is required because of the limited sample in the study.

The primary goal of our study is to propose an integrated AI-driven glioma molecular diagnostic workflow that not only complements but also enhances human pathologists’ diagnostic capabilities. This is particularly significant as the current approach of predicting molecular features solely from H&E-stained sections relies exclusively on human expertise. Rather than emphasizing the superiority of our approach over existing AI methods, we focused on validating its utility in a real-world setting by conducting a direct comparative study with human pathologists, who represent the current gold standard in this domain. Regarding comparative analyses with other AI methods, evaluations of several models for patch-level predictions were conducted but only marginal differences in performance were revealed in our study. While GLISP can be beneficial from conducting an exhaustive search of the pretrained models, such an approach may lead to overfitting given the limited sample size.

### 4.3. Technical Strengths and Challenges

From a technical standpoint, we present three important points of discussion. First, the present study utilized one image (instance)—one prediction scheme for patch-level CNN (GLISP-P), which may not provide adequate information about the WSI despite its good performance. To overcome this drawback, our MLP meta-model (GLISP-W) was rendered equivalent to the pooling function of MIL. In MIL, the model considers several instances (images and patches) as inputs and yields only one label, such as a genetic event (yes or no), gene expression (numeric value), or survival risk. We attention-pooled 32 encoded vectors from a WSI to simulate a small biopsy area (0.5 mm^2^), aiming to prevent overfitting in IDH mutation prediction on MVP and necrosis patches. Second, the sample size was relatively small, leading to possible population-based genetic biases. Therefore, the problem of out-of-distribution (OOD) data is still possible and can be serious in outside cases, given the significant variation in the histological features of adult glioma and tissue processing in separate institutes. Even with the many regularization strategies used in the present study, larger data with various morphologies is the key to training a model that can be reliable in pathology practice. Third, our model represents a composable framework for predicting frequent genetic events in gliomas, image level, and slide level (GLISP-P and GLISP-W). GLISP-P is a small CNN for a single genetic event, therefore, training and interpreting the output is easy. GLISP-P and GLISP-W could be fine-tuned separately for OOD data. GLISP is flexible in incorporating novel genetic events expected by the WHO 2021 or later.

### 4.4. Future Directions

There are several improvements that could be made in our future studies with larger training samples with more availability of all interested genetic events. GLISP-P may need to be scaled up with respect to width and depth while GLISP-W may require a more sophisticated design. First, GLISP-P could be built up with more convolutional blocks and layers or replaced by more sophisticated and complex models, although a pilot study to select the optimal models is required. Second, a more sophisticated design of GLISP-W, such as multi-gene cross-attention, can be designed to leverage the mutual information between genetic events, providing a more comprehensive and interpretable result of gene prediction.

### 4.5. Conclusions

The proposed GLISP framework is a compatible approach with a composable nature for predicting genetic events in adult gliomas in accordance with WHO 2021 classification. This architecture can predict the genetic events in patch-level and WSI-level inputs.

## Figures and Tables

**Table 1:** Clinicopathological information of patients in TWMU dataset.

		Astrocytoma	Oligodendroglioma	Glioblastoma	*p*-Value
Patient number		38	40	30	
Gender	Male/Female	25/13	29/11	18/12	0.542
Age	Mean (range)	41.5 (22–64)	43.7 (24–67)	62.3 (32–82)	0.107
Location	Frontal	23	30	16	0.135
	Temporal	14	6	11	
	Parietal	1	4	2	
	Occipital	0	0	1	
CNS WHO grade	2	11	22	0	<0.001
	3	20	18	0	
	4	7	0	30	
IDH and 1p/19q status	IDH-wt	0	0	30	<0.001
	IDH-mt and codel (+)	0	40	0	
	IDH-mt and codel (−)	38	0	0	

**Table 2:** Cross-validation results of GLISP.

Patch-Level Prediction	ROC-AUC	Accuracy	F1	Precision	Recall
*IDH1/2* mutation	0.75 (±0.028)	0.72 (±0.039)	0.62 (±0.030)	0.59 (±0.037)	0.68 (±0.106)
*TP53* mutation	0.70 (±0.034)	0.66 (±0.033)	0.58 (±0.013)	0.60 (±0.048)	0.57 (±0.030)
*ATRX* mutation	0.63 (±0.090)	0.57 (±0.112)	0.49 (±0.072)	0.40 (±0.101)	0.69 (±0.146)
*TERT* promoter mutation	0.67 (±0.079)	0.63 (±0.062)	0.63 (±0.062)	0.66 (±0.047)	0.54 (±0.148)
*EGFR* amplification	0.73 (±0.030)	0.69 (±0.036)	0.51 (±0.040)	0.40 (±0.037)	0.69 (±0.057)
*CDKN2A/B* homo. del.	0.69 (±0.026)	0.65 (±0.048)	0.51 (±0.041)	0.45 (±0.063)	0.64 (±0.130)
7 gain/10 loss	0.75 (±0.028)	0.72 (±0.039)	0.62 (±0.030)	0.59 (±0.037)	0.68 (±0.106)
1p/19q codeletion	0.73 (±0.039)	0.68 (±0.048)	0.49 (±0.050)	0.38 (±0.054)	0.70 (±0.073)
*MGMT* promoter meth.	0.64 (±0.052)	0.59 (±0.060)	0.66 (±0.078)	0.82 (±0.046)	0.56 (±0.119)
**WSI-Level Prediction**	**ROC-AUC**	**Accuracy**	**F1**	**Precision**	**Recall**
*IDH1/2* mutation	0.79 (±0.039)	0.74 (±0.045)	0.68 (±0.045)	0.59 (±0.038)	0.79 (±0.090)
*TP53* mutation	0.70 (±0.029)	0.70 (±0.021)	0.64 (±0.030)	0.63 (±0.064)	0.67 (±0.098)
*ATRX* mutation	0.65 (±0.109)	0.63 (±0.139)	0.54 (±0.088)	0.46 (±0.115)	0.70 (±0.152)
*TERT* promoter mutation	0.73 (±0.083)	0.71 (±0.052)	0.64 (±0.081)	0.82 (±0.101)	0.55 (±0.133)
*EGFR* amplification	0.78 (±0.029)	0.73 (±0.054)	0.57 (±0.044)	0.45 (±0.097)	0.80 (±0.105)
*CDKN2A/B* homo. del.	0.74 (±0.032)	0.66 (±0.059)	0.57 (±0.029)	0.47 (±0.091)	0.80 (±0.152)
7 gain/10 loss	0.79 (±0.039)	0.74 (±0.045)	0.68 (±0.045)	0.59 (±0.038)	0.79 (±0.090)
1p/19q codeletion	0.80 (±0.052)	0.73 (±0.065)	0.56 (±0.075)	0.45 (±0.123)	0.81 (±0.092)
*MGMT* promoter meth.	0.67 (±0.074)	0.59 (±0.085)	0.63 (±0.111)	0.87 (±0.036)	0.51 (±0.127)

**Table 3:** Confusion Matrix of Molecular Diagnosis between GLISP-predicted Diagnosis and ground-truth Diagnosis in TWMU dataset.

		Ground-Truth Diagnosis		
		IDH-Mutant Astrocytoma	Oligodendroglioma	IDH-Wildtype Glioblastoma
GLISP-predicted diagnosis	IDH-mutant Astrocytoma	29	2	7
	Oligodendroglioma	9	19	12
	IDH-wildtype Glioblastoma	7	0	23

## Data Availability

The data and codes are preserved and under improvement for the release in future projects.

## Supplementary Materials

The following supporting information can be downloaded at: https://www.mdpi.com/article/10.3390/bioengineering12010012/s1, Supplementary Files.pdf, consisting of 4 parts. Part 1 (Figure S1). Detailed architectures of GLISP-P and GLISP-W. Part 2 (Figures S2–S10). Full results of cross-validation ROC analyses of GLISP’s predictions of nine genetic events. Part 3 (10 sub-figures). Full results of GLISP vs. Pathologists comparison. Part 4 (Figures S11–S19). Pixel-level DeepLIFT heatmaps of predictions of nine genetic events.
