# Supplementary material for: Glioma Image-Level and Slide-Level Gene Predictor (GLISP) for Molecular Diagnosis and Predicting Genetic Events of Adult Diffuse Glioma
Source: Bioengineering (Basel). 2024 Dec 27;12(1):12. doi: 10.3390/bioengineering12010012 (PMC11761954; doi:10.3390/bioengineering12010012)

Part 1. GLISP architecture

Figure S1. Detailed architectures of GLISP-P and GLISP-W

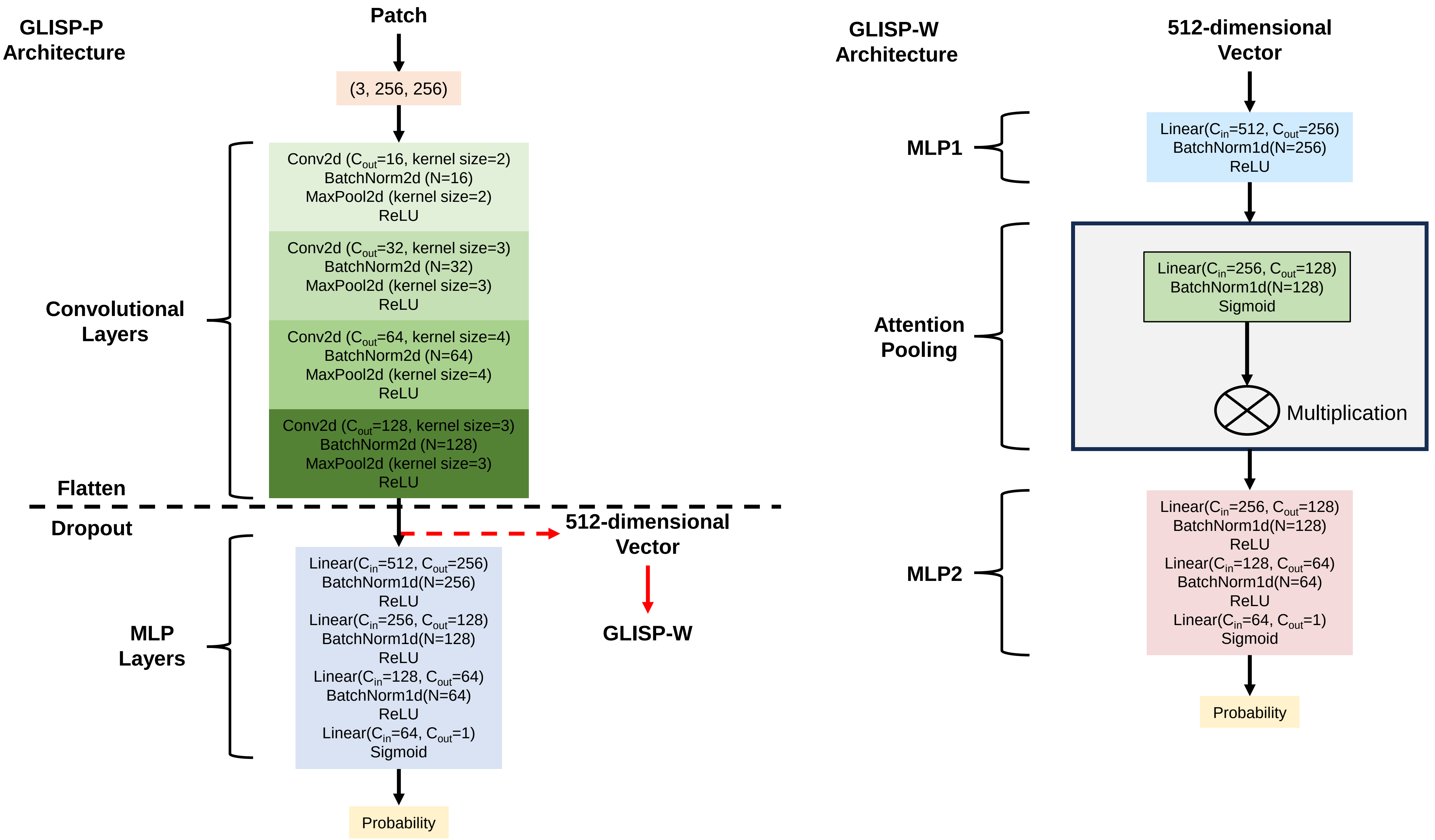

Part 2. Cross-validation Results

Figure S2.
ATRX

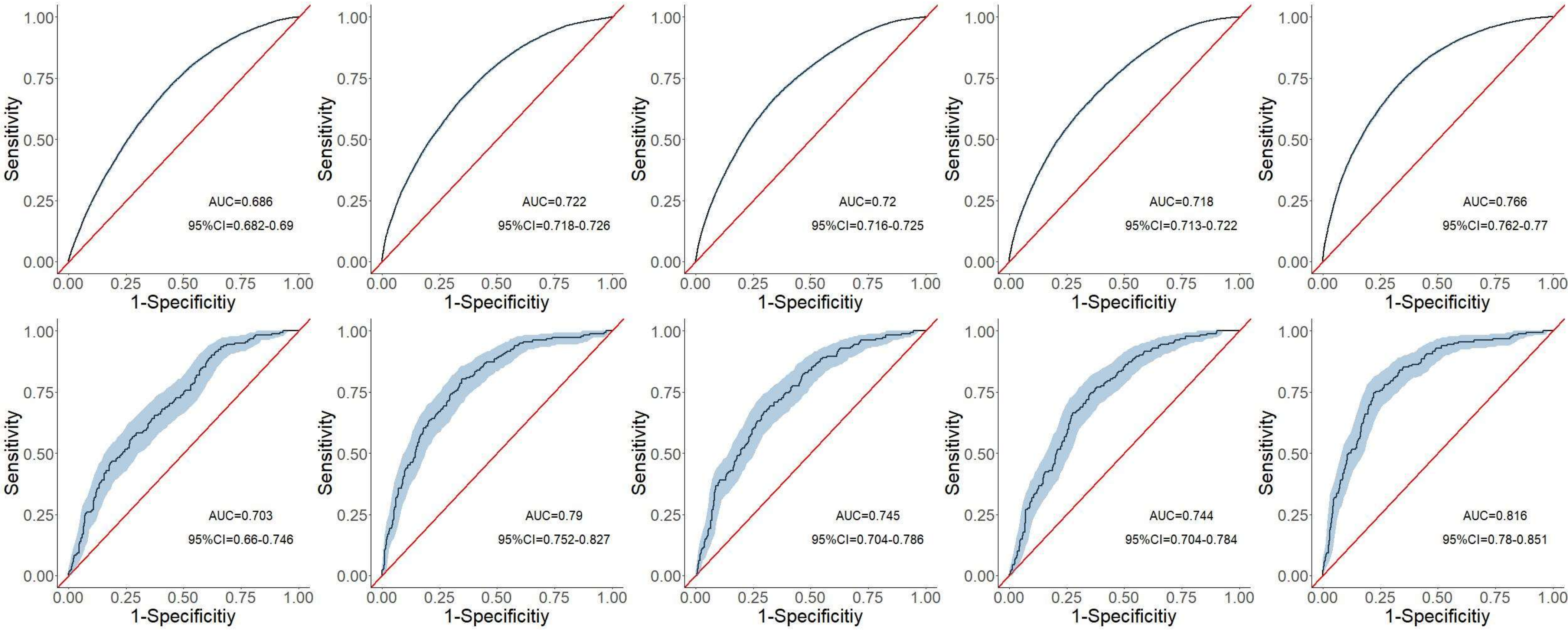

Figure S3.
***CDKN2A/B* homozygous deletion**

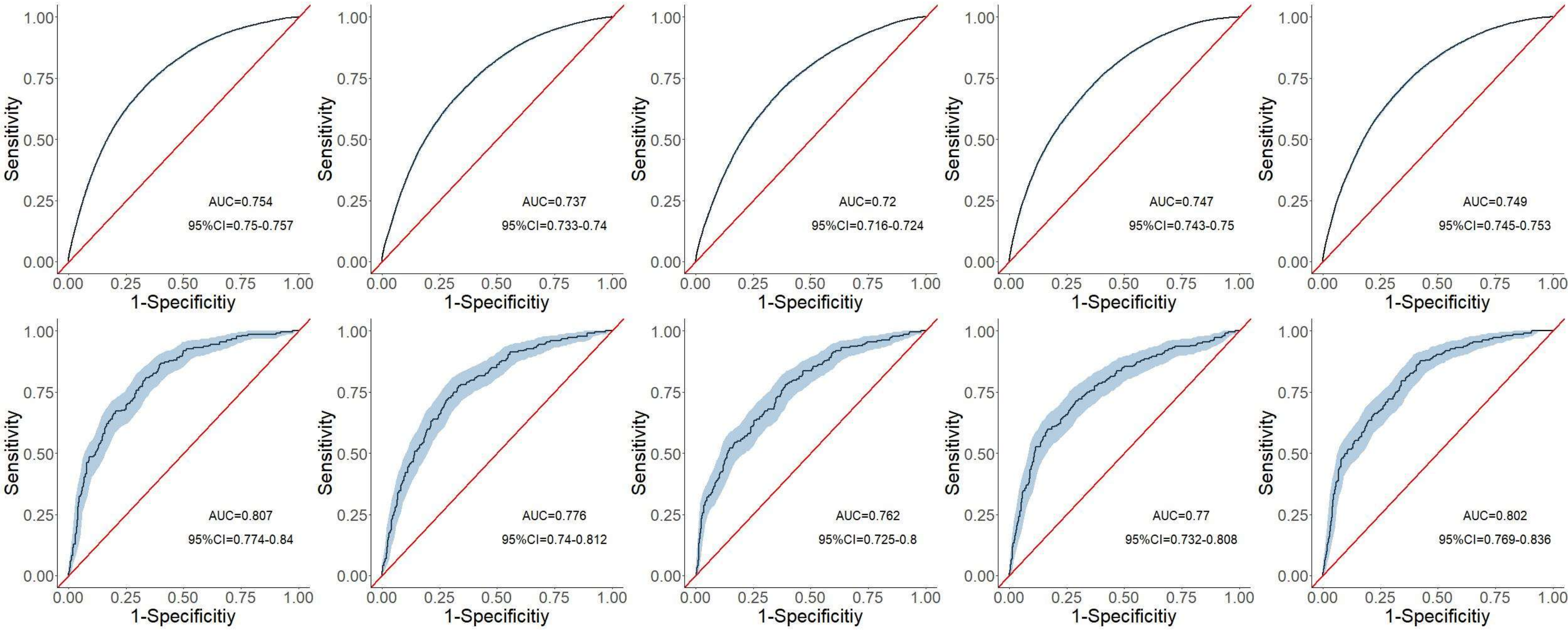

Figure S4.
1p/19q co-deletion

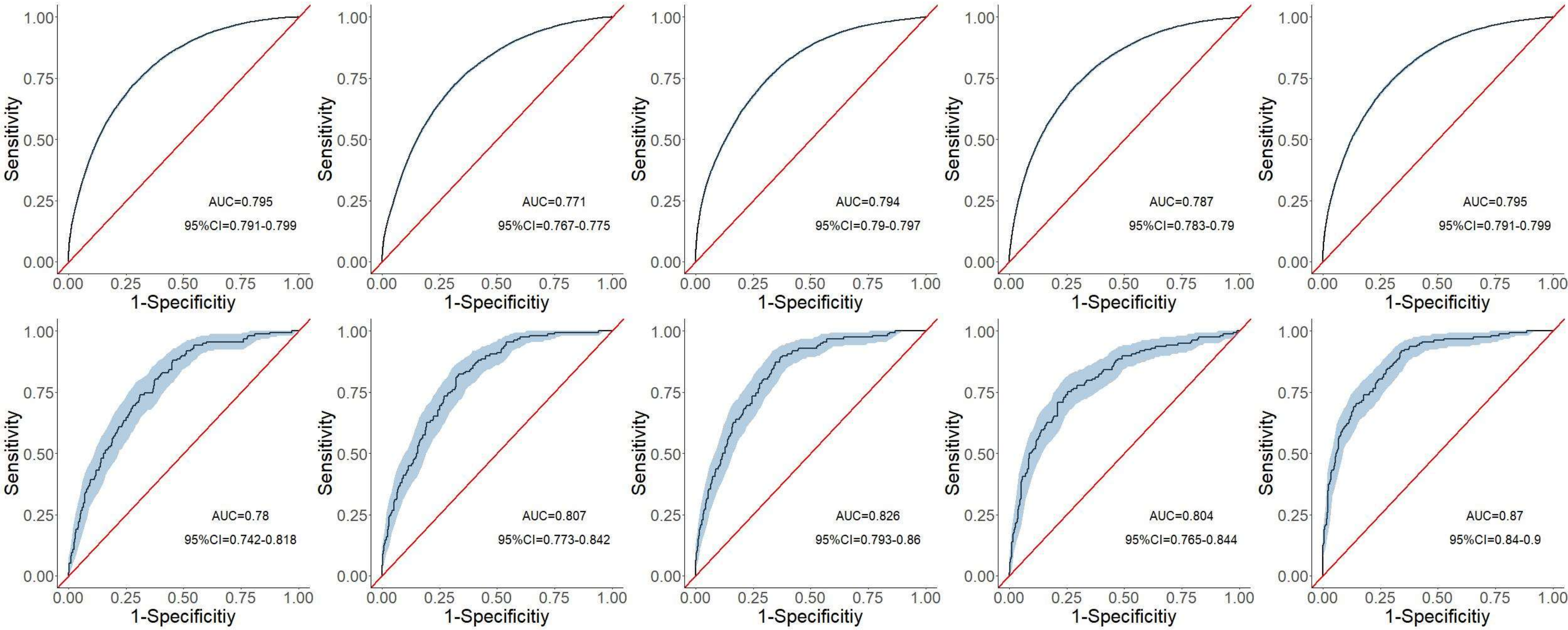

Figure S5.
***EGFR* amplification**

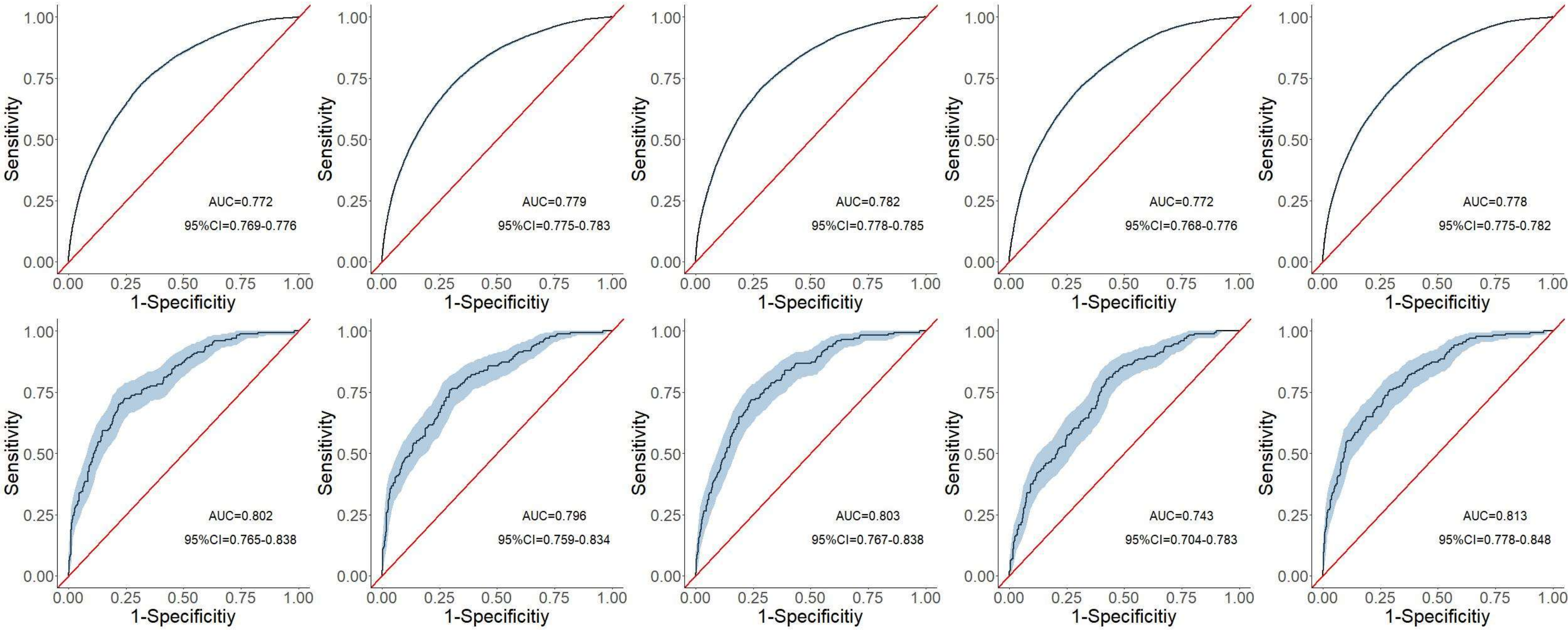

Figure S6.
7+/10-

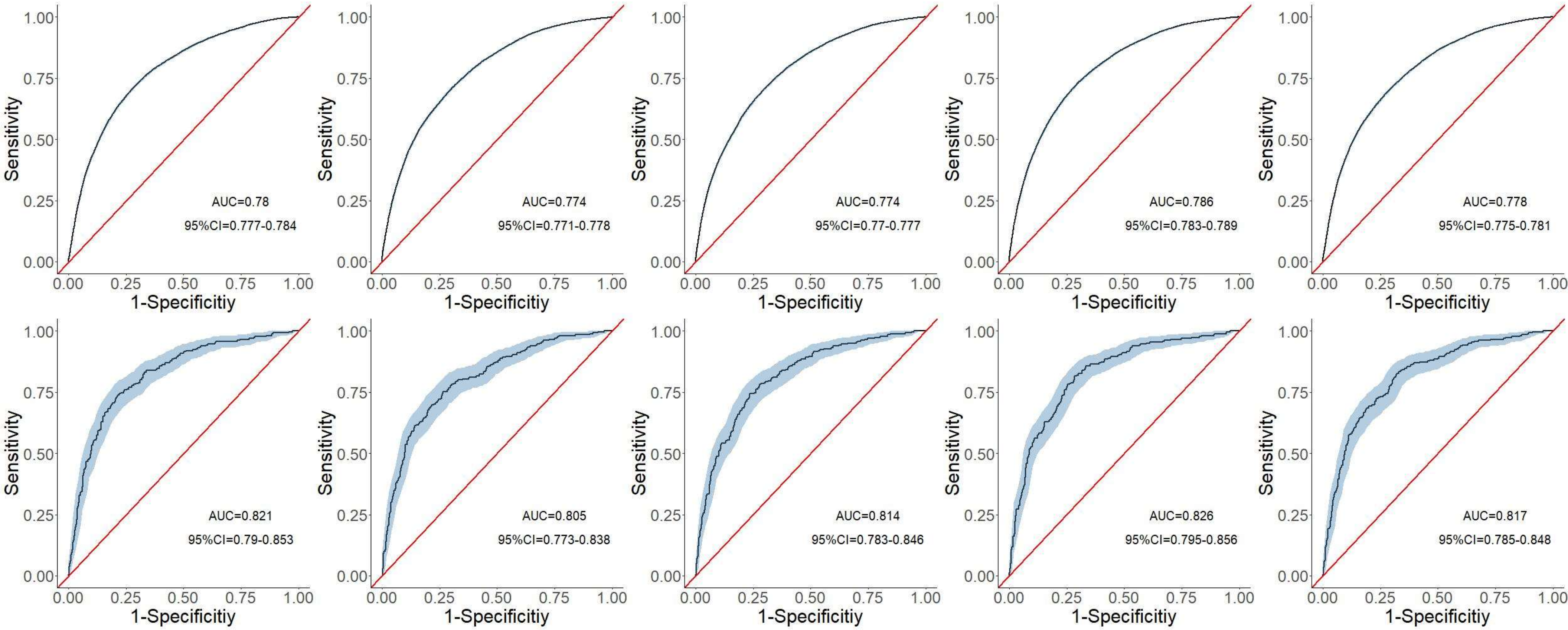

Figure S7.
***IDH1/2* mutation**

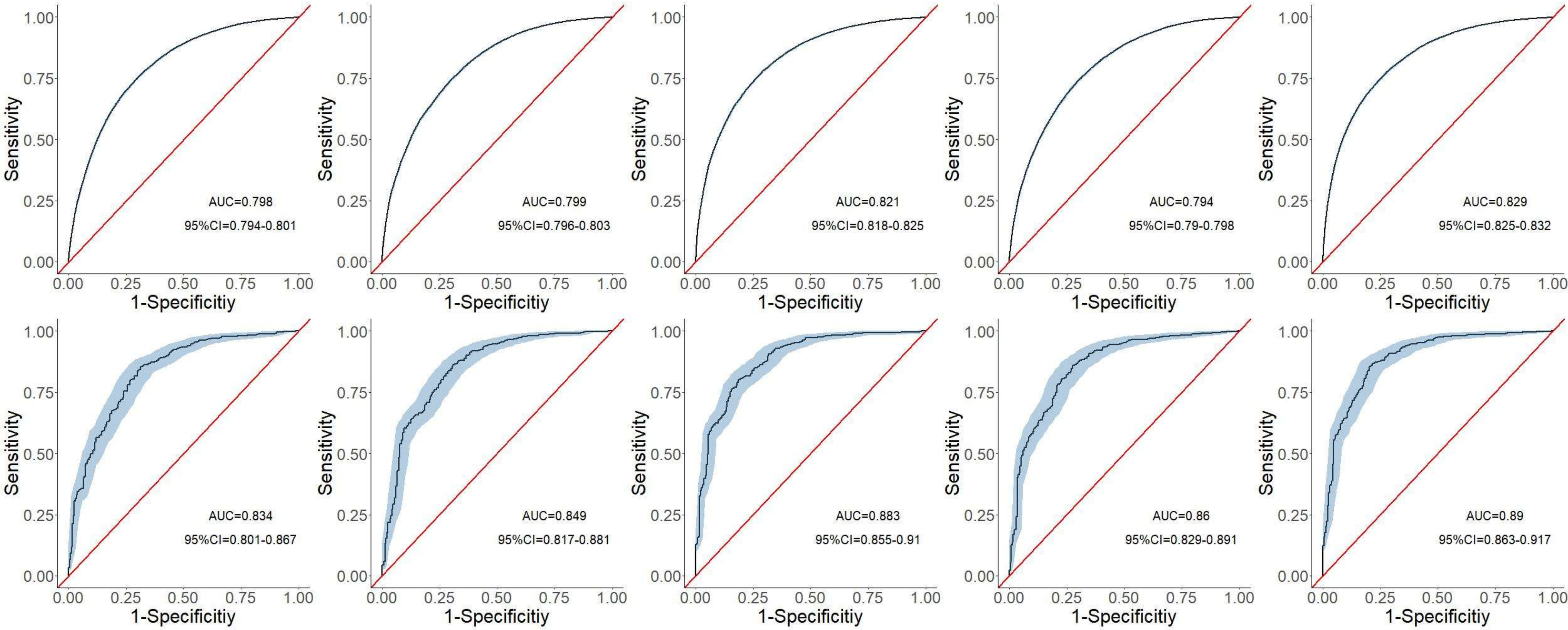

Figure S8.
***MGMT* promoter hypermethylation**

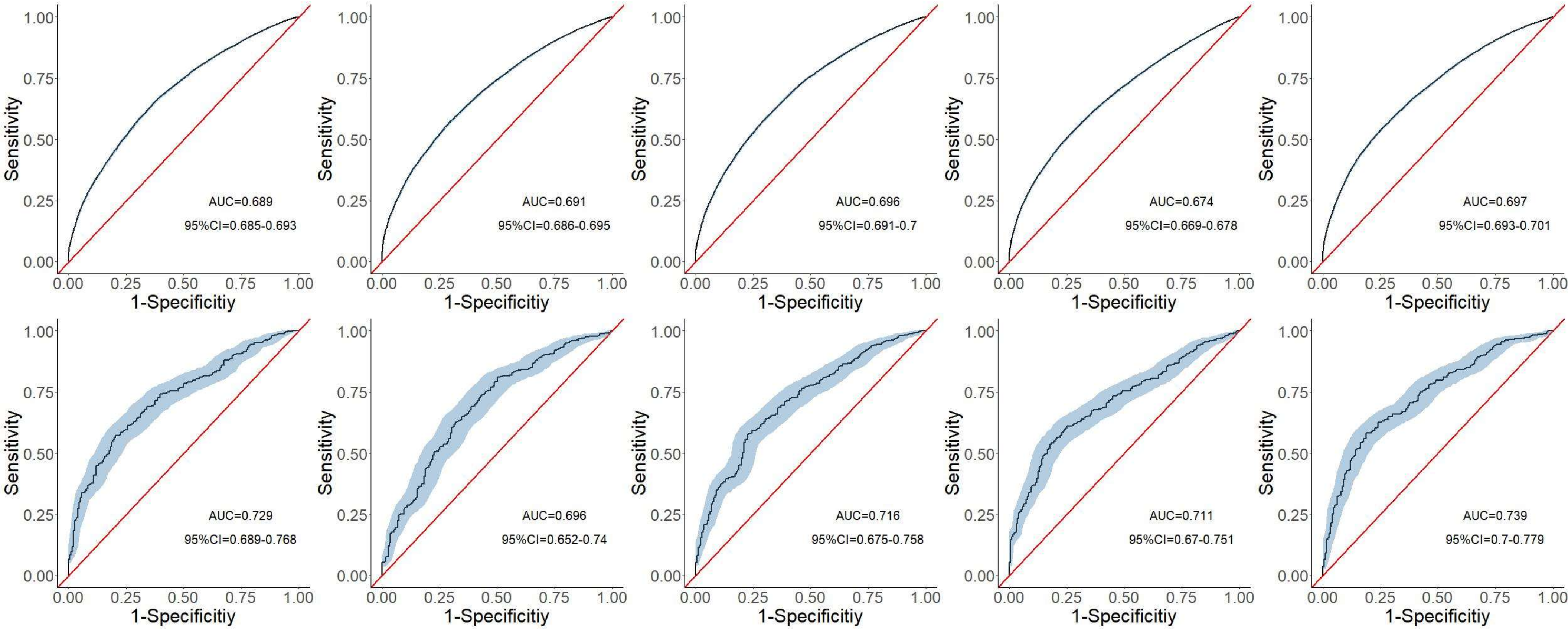

Figure S9.
***TERT* promoter mutation**

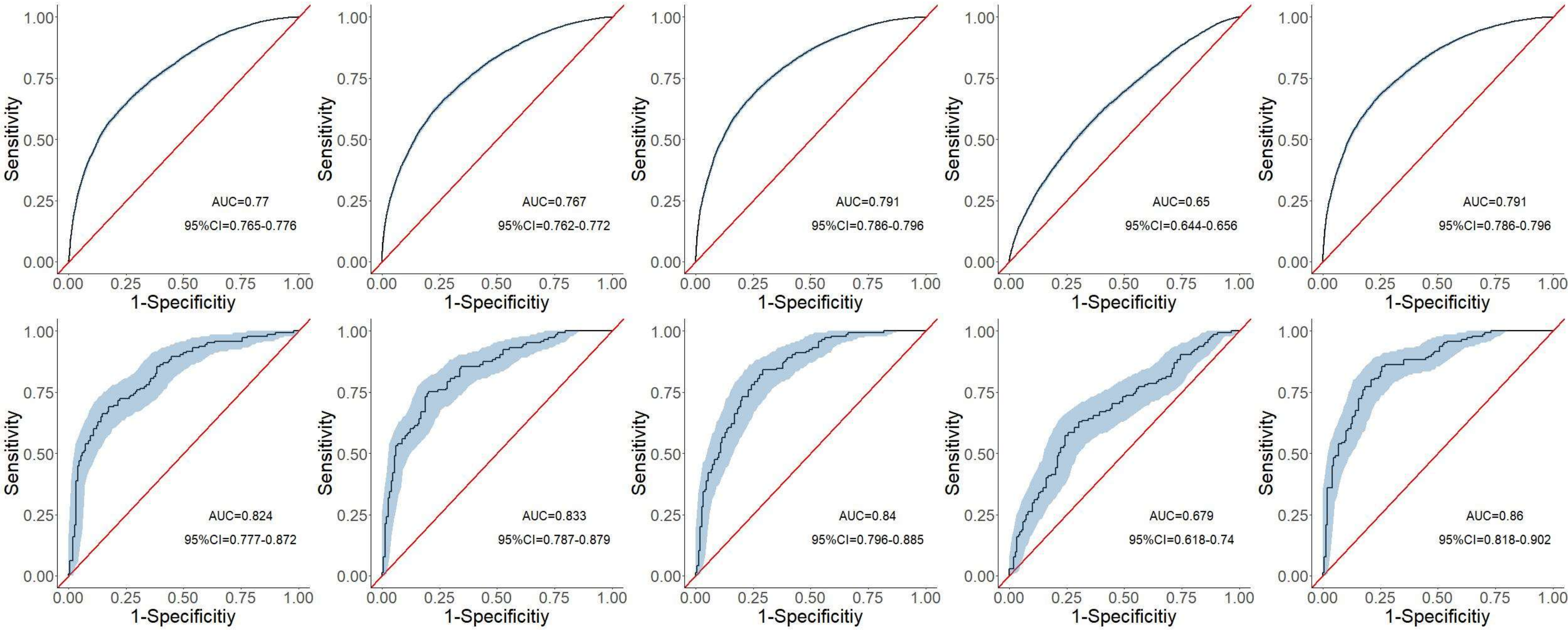

Figure S10.
***TP53* mutation**

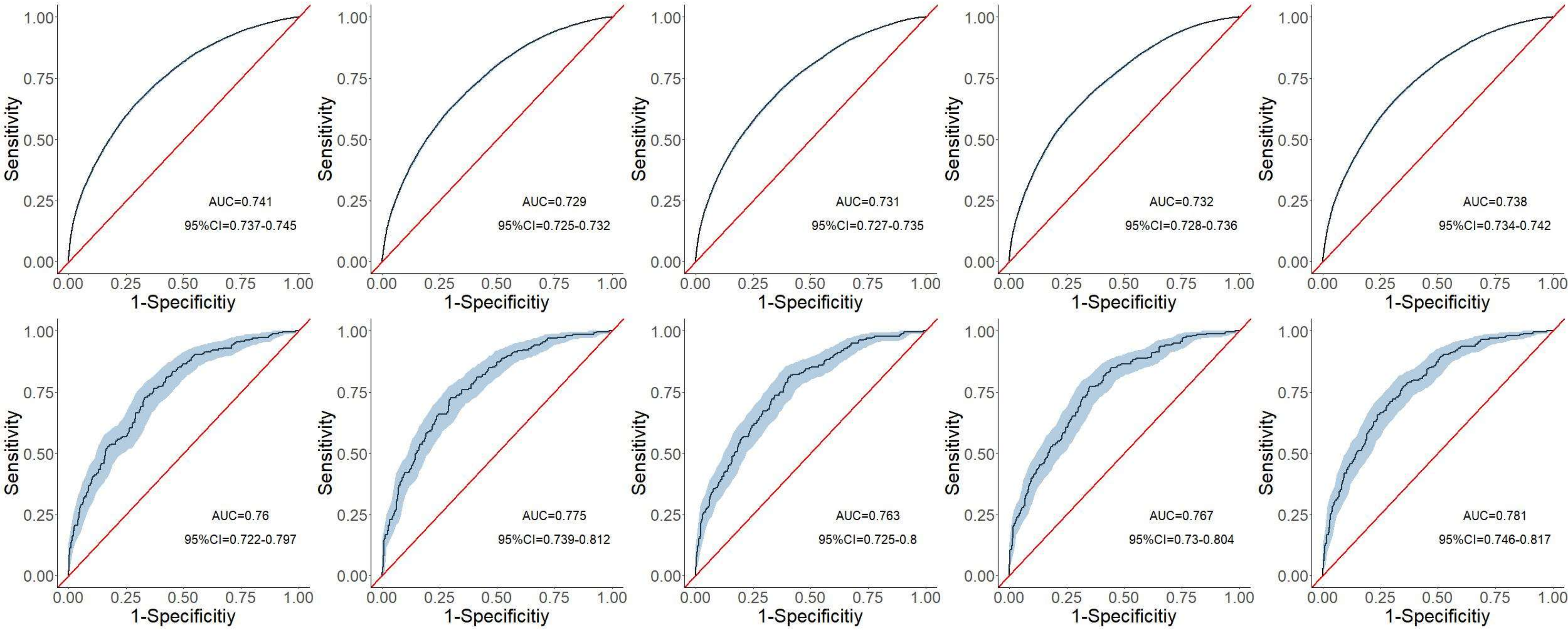

Part 3. GLISP vs. Pathologists

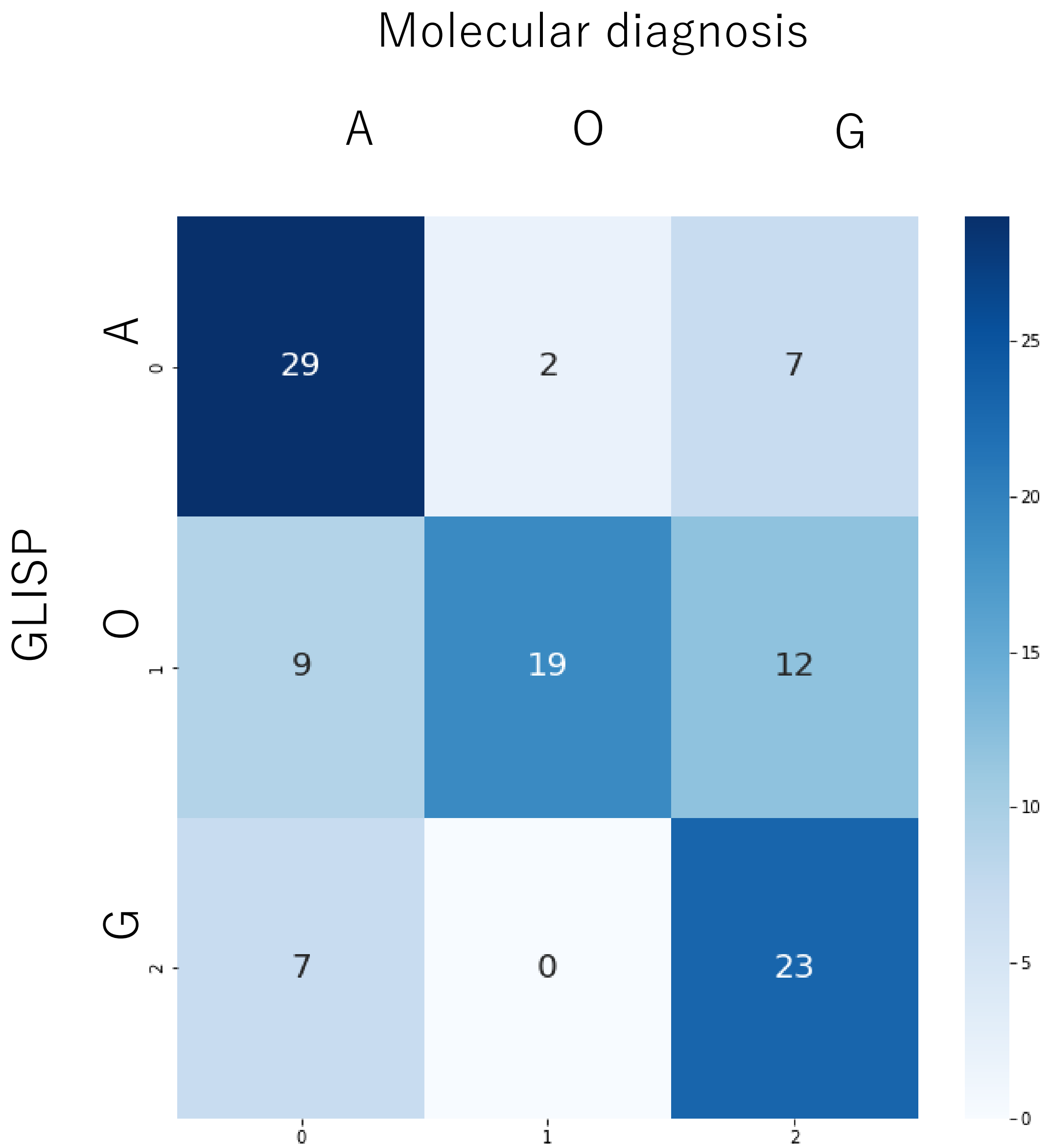

Accuracy: 0.66

Macro averaged F1 score: 0.65

Weighted F1 score: 0.65

sensitivity/specificity
Astrocytoma: 0.76 / 0.60
Oligodendroglioma: 0.48 / 0.76
Glioblastoma : 0.77 / 0.62

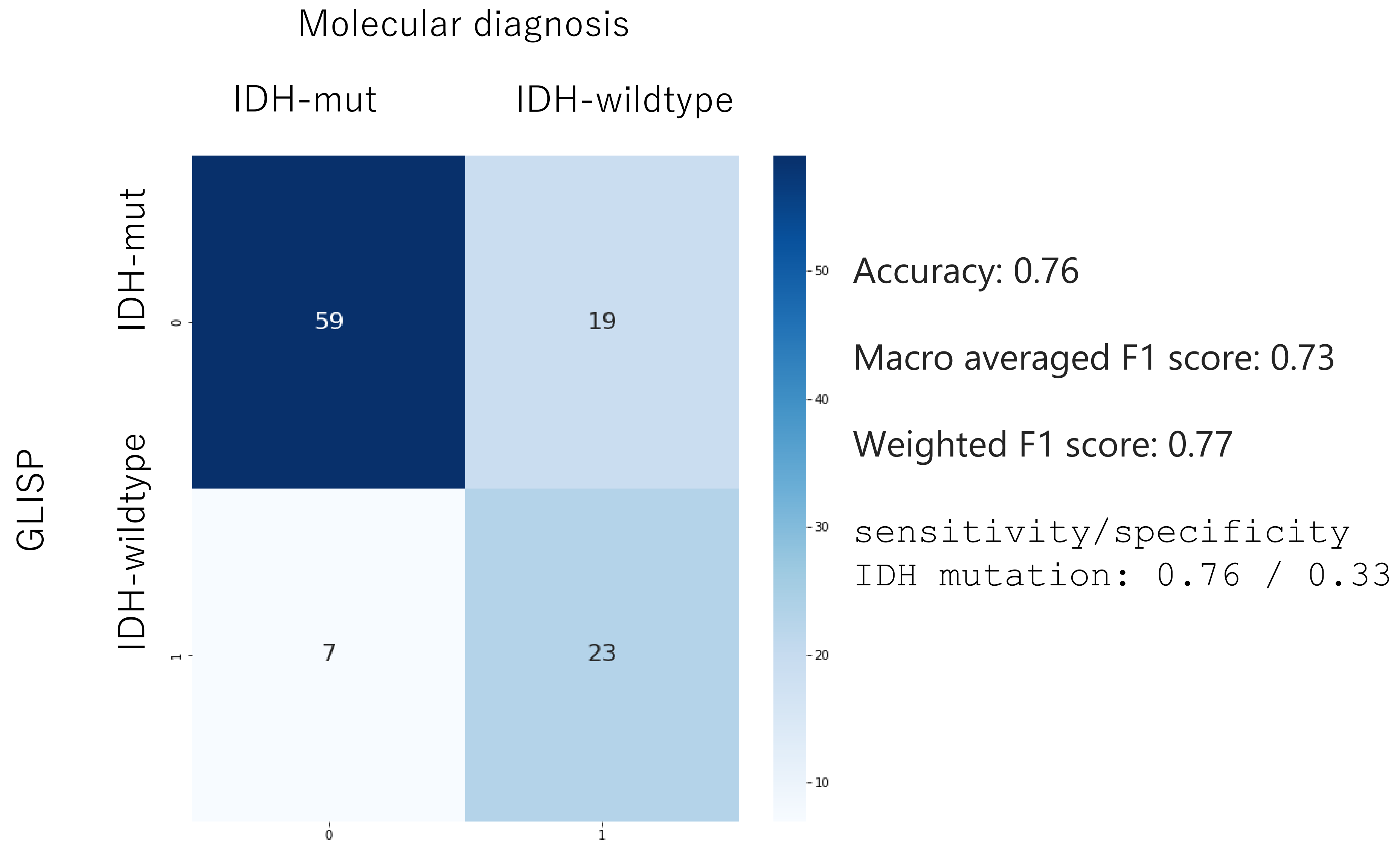
Molecular diagnosis
IDH-mut
IDH-wildtype
GLISP
IDH-mut
IDH-wildtype
59
19
7
23
0
1
0
1
50
40
30
20
10
Accuracy: 0.76
Macro averaged F1 score: 0.73
Weighted F1 score: 0.77
sensitivity/specificity
IDH mutation: 0.76 / 0.33

Human pathologist 1(T.I.)

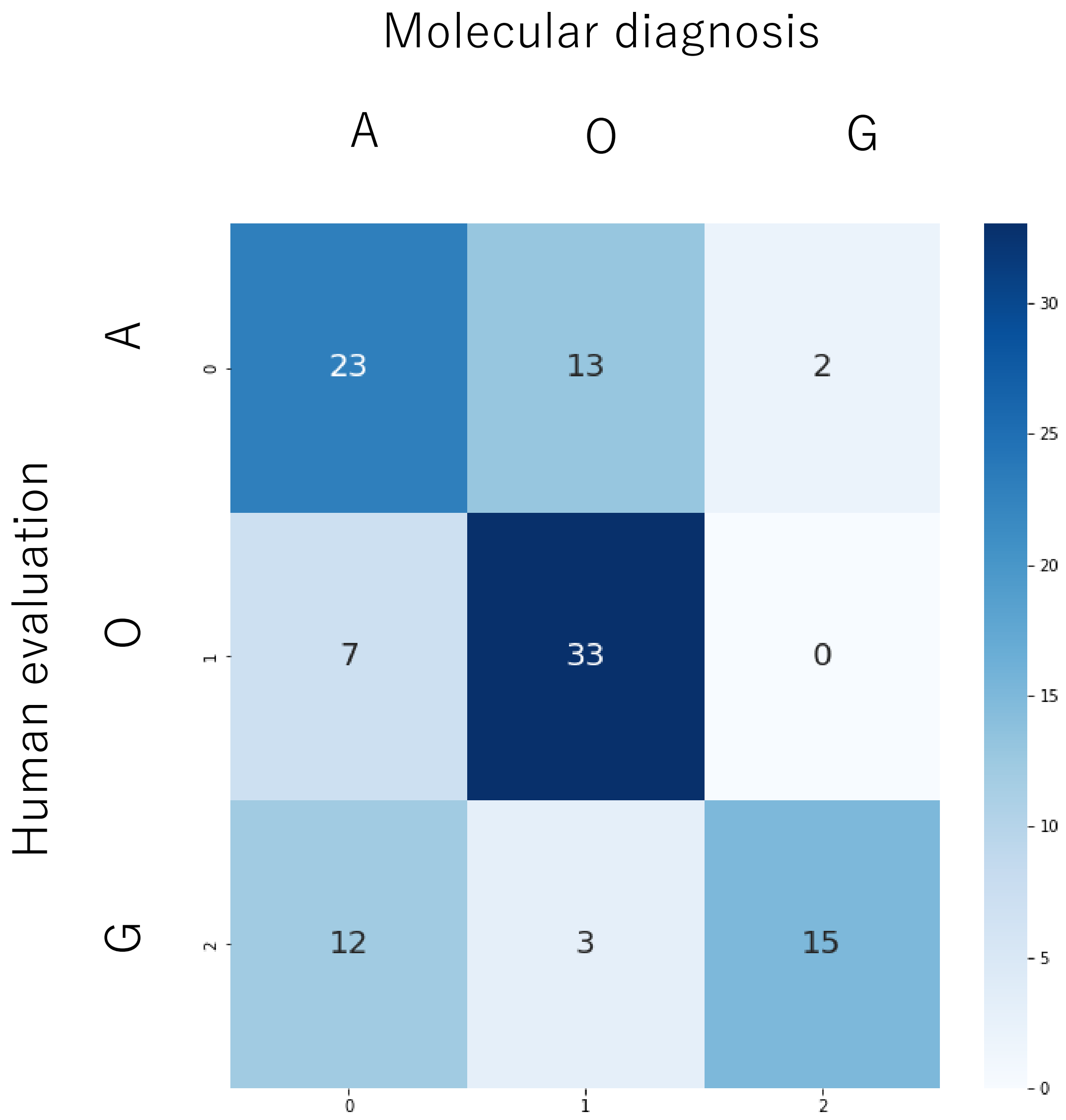

Accuracy: 0.66

Macro averaged F1 score: 0.65

Weighted F1 score: 0.65

sensitivity/specificity
Astrocytoma: 0.61 / 0.69
Oligodendroglioma: 0.83 / 0.56
Glioblastoma: 0.50 / 0.72

Human pathologist 1(T.I.)

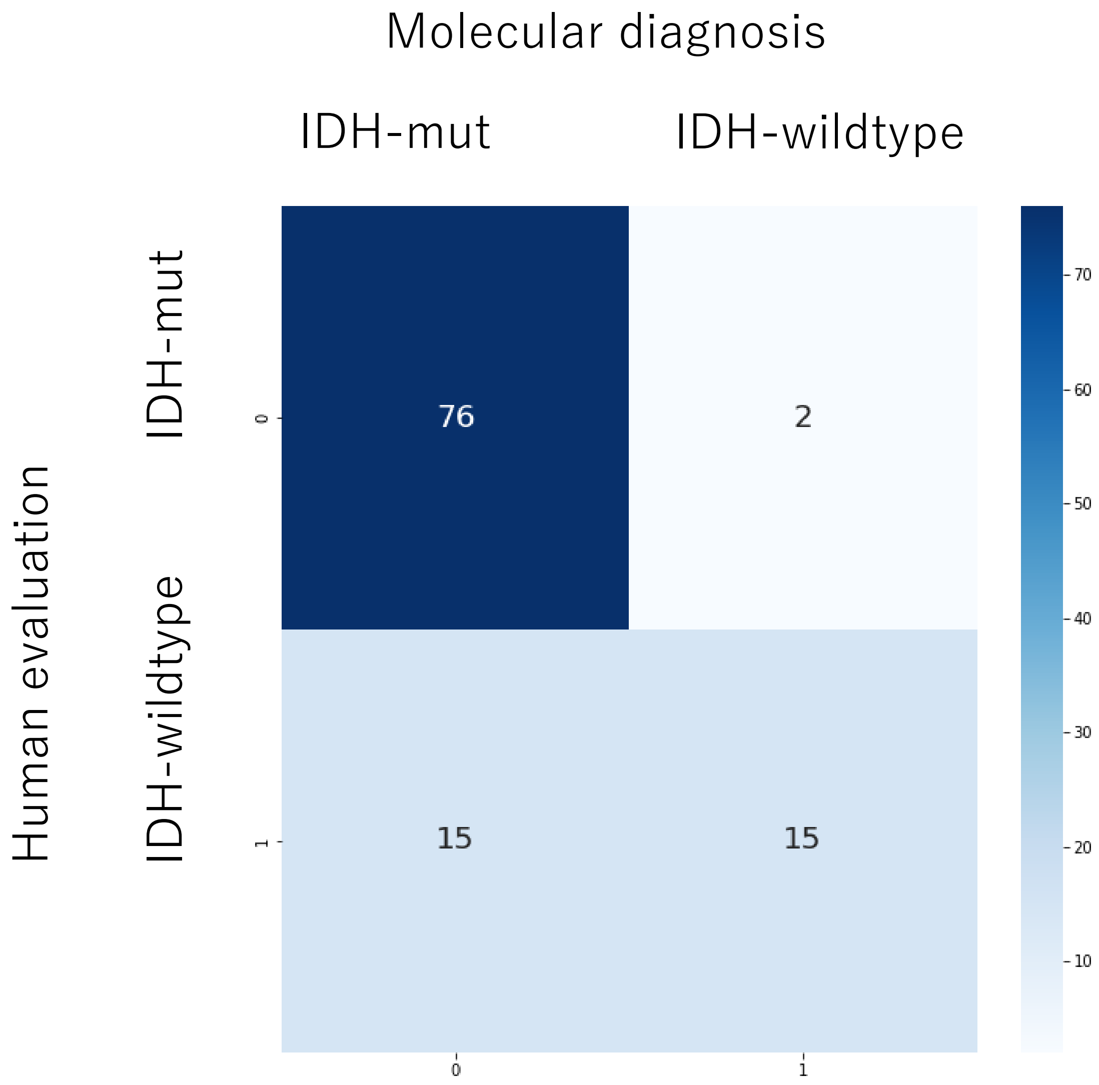

Accuracy: 0.84

Macro averaged F1 score: 0.77

Weighted F1 score: 0.83

sensitivity/specificity
IDH mutation: 0.97 / 0.21

Human pathologist 2(I.T.)

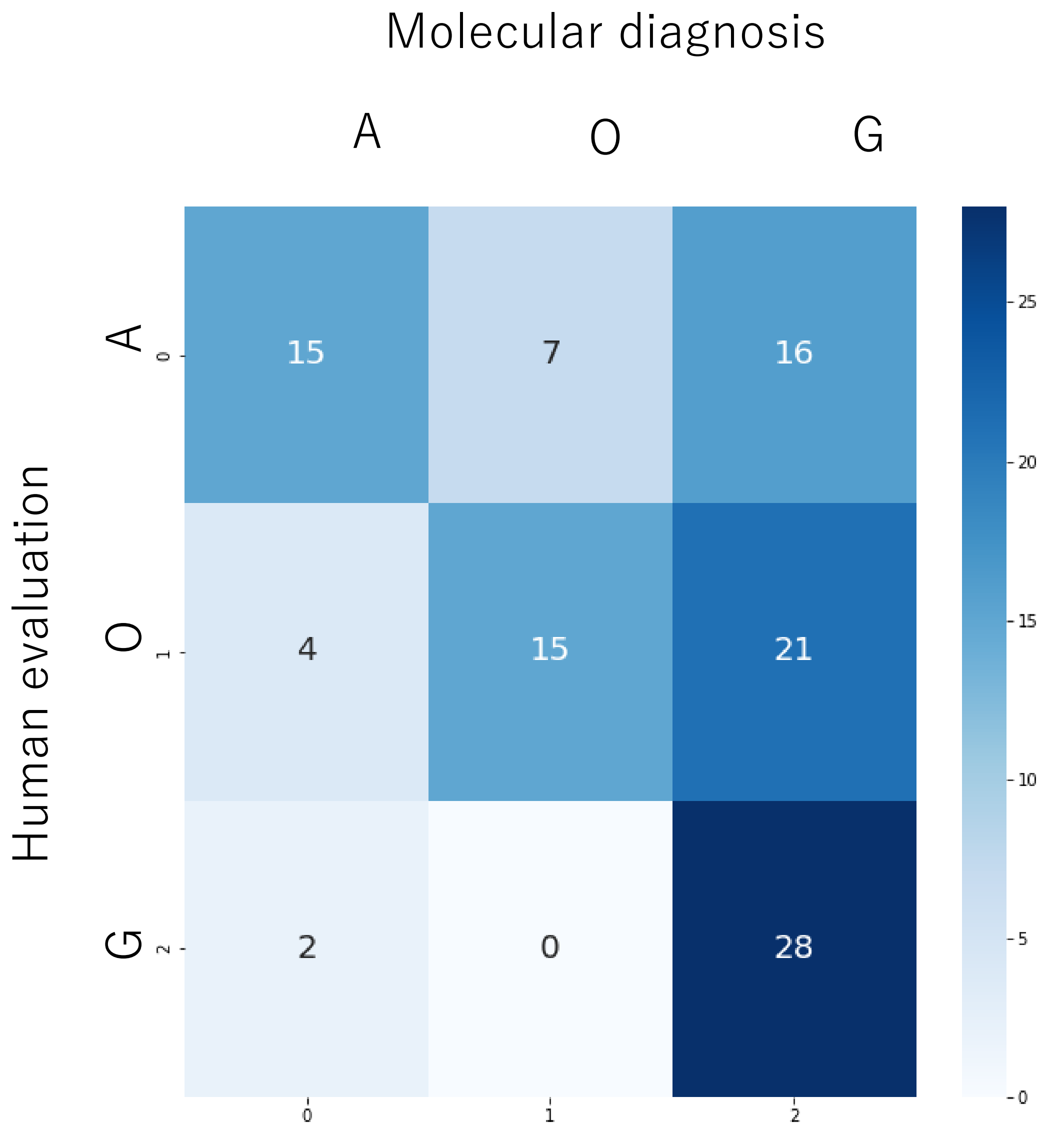

Accuracy: 0.54

Macro averaged F1 score: 0.53

Weighted F1 score: 0.52

```
sensitivity/specificity
Astrocytoma: 0.39 / 0.61
Oligodendroglioma: 0.38 / 0.63
Glioblastoma: 0.93 / 0.38
```

Human pathologist 2(I.T.)

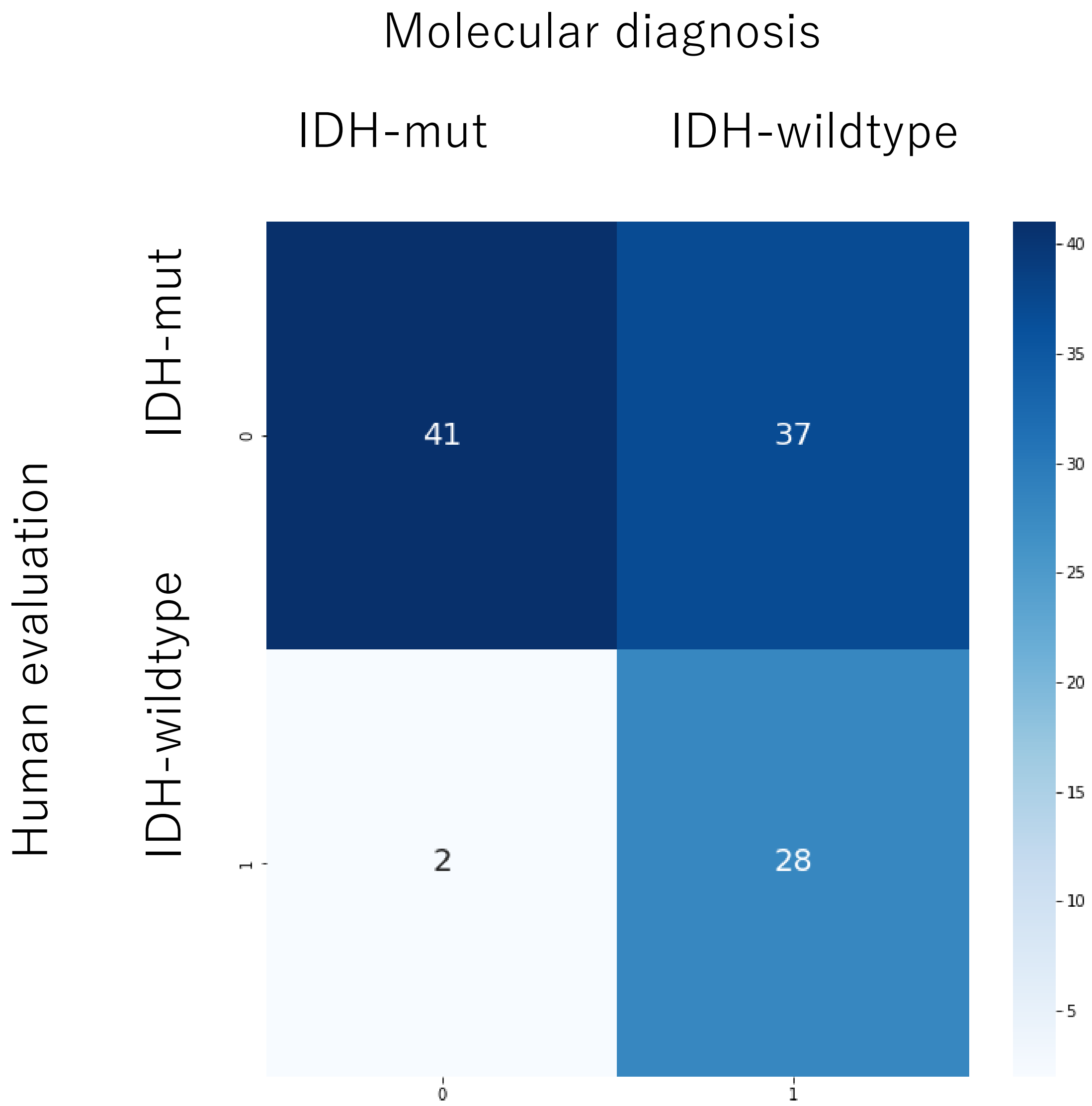

Accuracy: 0.64

Macro averaged F1 score: 0.63

Weighted F1 score: 0.65

sensitivity/specificity
IDH mutation: 0.53 / 0.40

Human pathologist 3(K.K.)

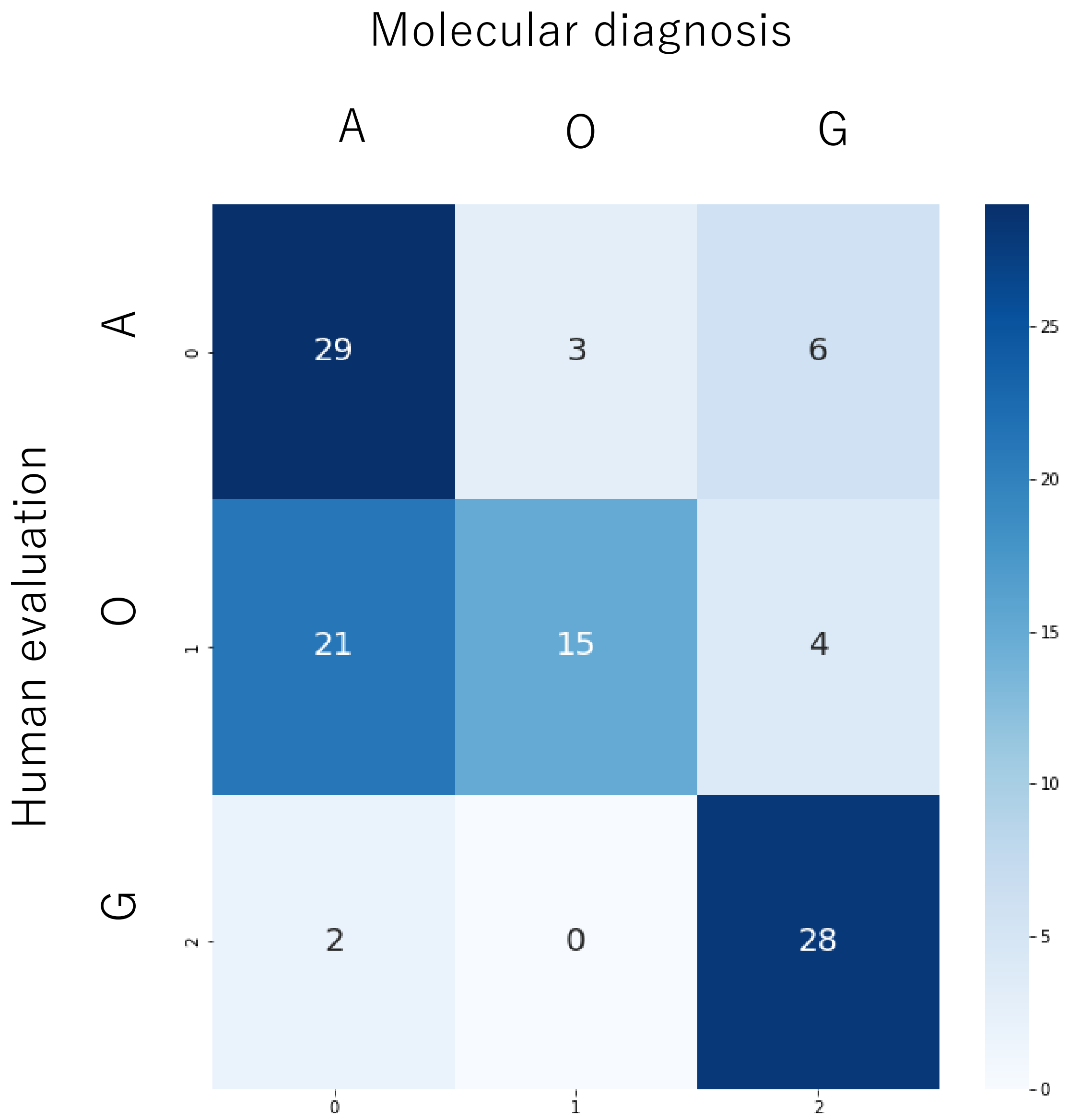

Accuracy: 0.67

Macro averaged F1 score: 0.66

Weighted F1 score: 0.65

sensitivity/specificity
Astrocytoma: 0.76 / 0.61
Oligodendroglioma: 0.38 / 0.84
Glioblastoma: 0.93 / 0.56

Human pathologist 3(K.K.)

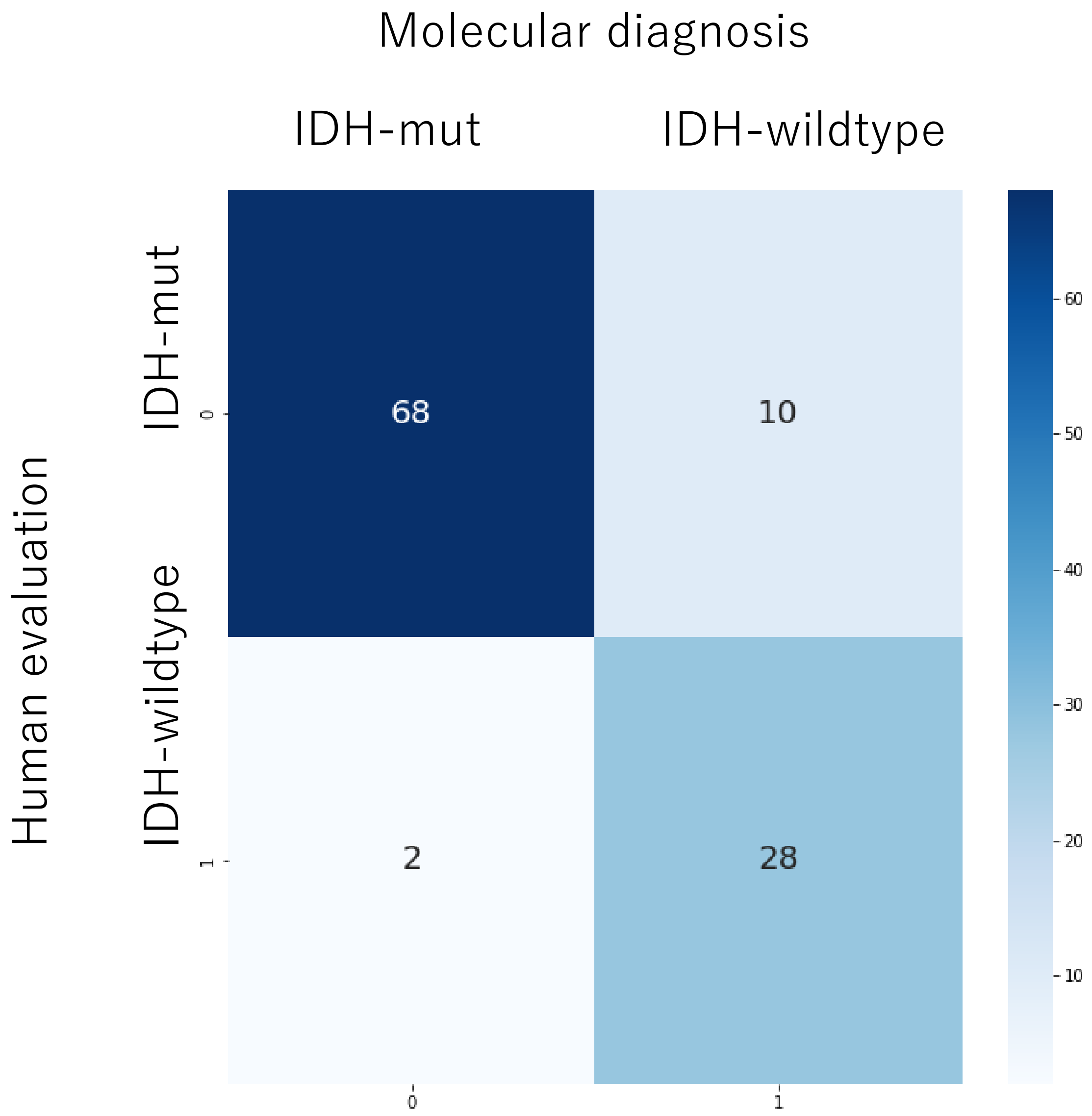

Accuracy: 0.89

Macro averaged F1 score: 0.87

Weighted F1 score: 0.89

sensitivity/specificity
IDH mutation: 0.87 / 0.40

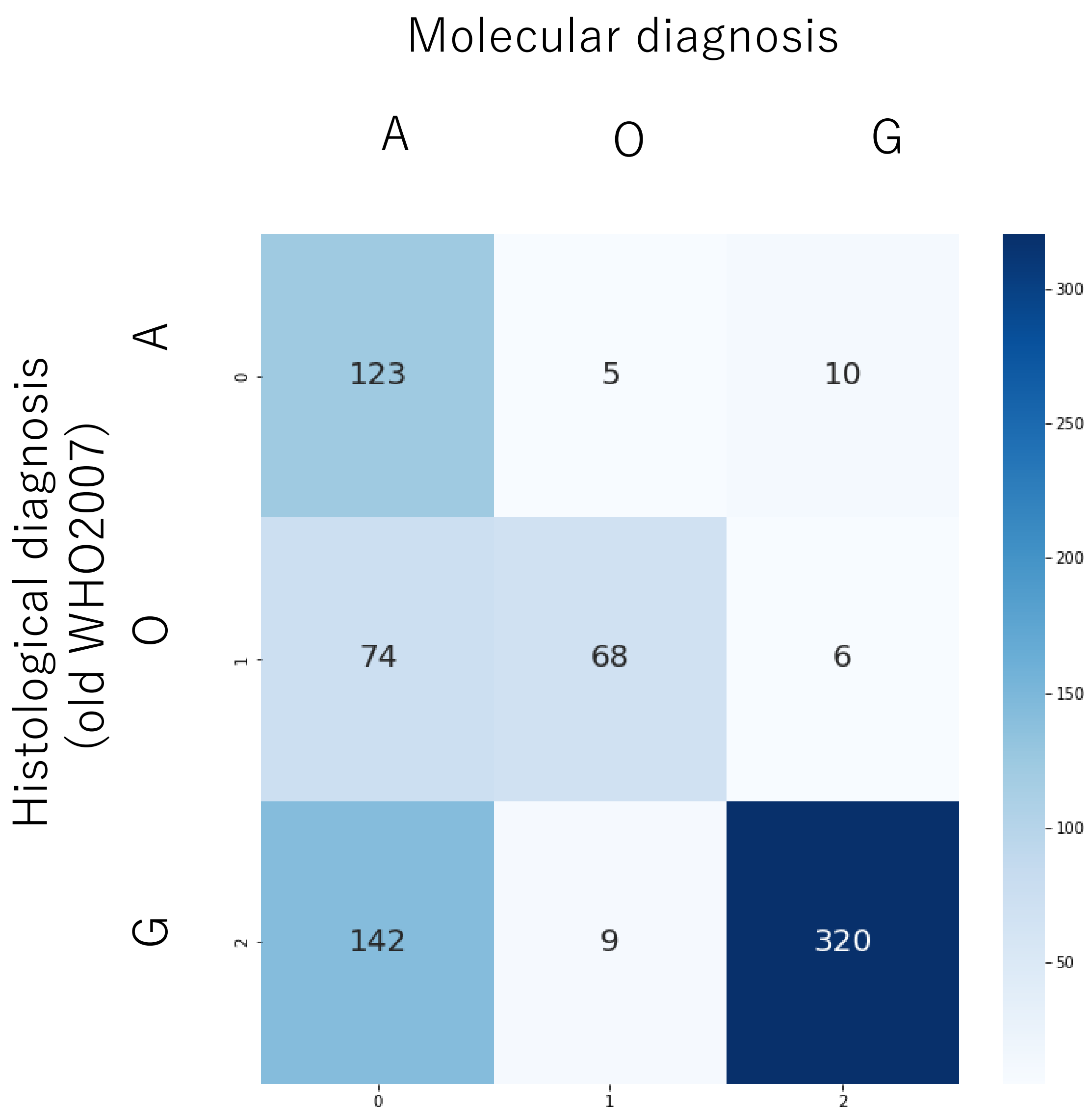

Accuracy: 0.68

Macro averaged F1 score: 0.63

Weighted F1 score: 0.70

sensitivity/specificity
Astrocytoma: 0.89 / 0.63
Oligodendroglioma: 0.46 / 0.73
Glioblastoma: 0.68 / 0.67

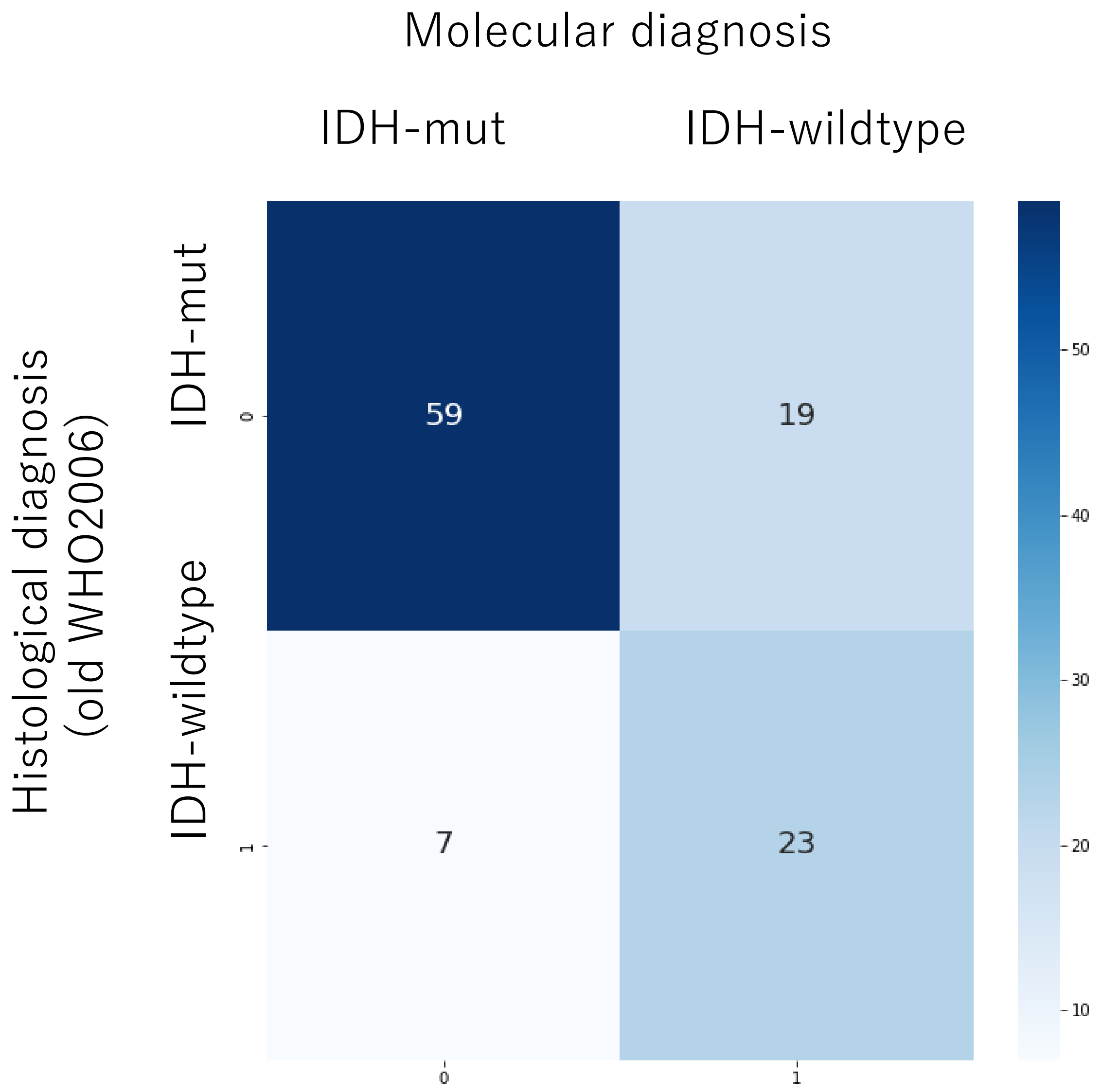

Accuracy: 0.78

Macro averaged F1 score: 0.78

Weighted F1 score: 0.78

sensitivity/specificity
IDH mutation: 0.94 / 0.52

Part 4. Model Interpretation

Figure S11. *ATRX* mutation. (A) the t-SNE plot of 1000 random patches (B) An example of pixel-level DeepLift heatmap.

B

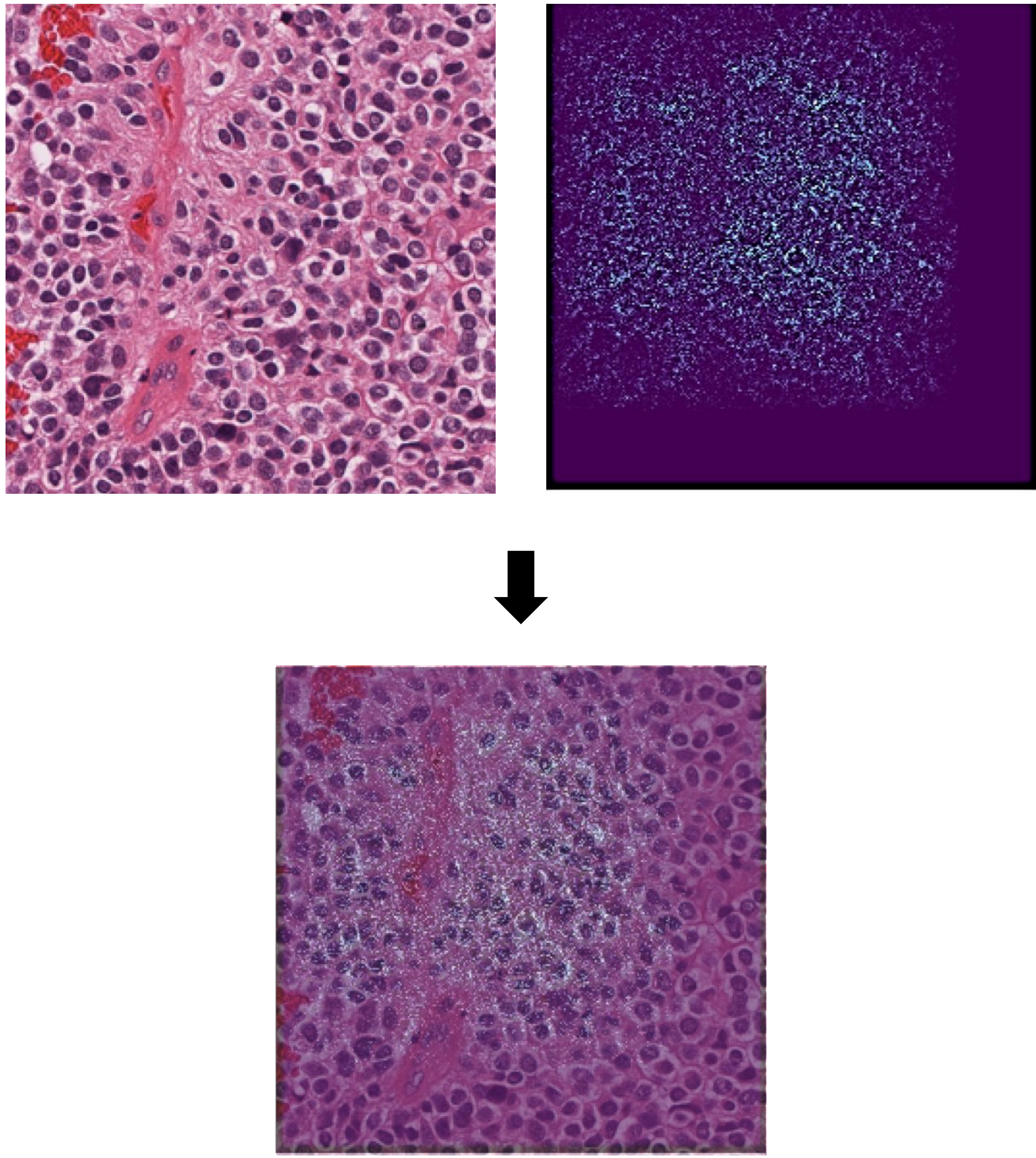

A

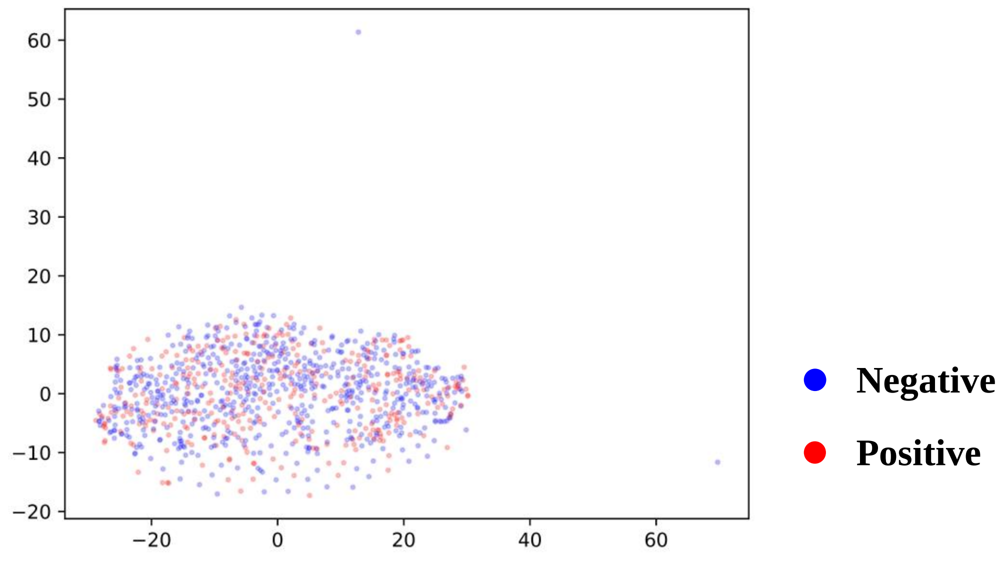

Figure S12. *CDKN2A/B* homozygous deletion. (A) the t-SNE plot of 1000 random patches (B) An example of pixel-level DeepLift heatmap.

A

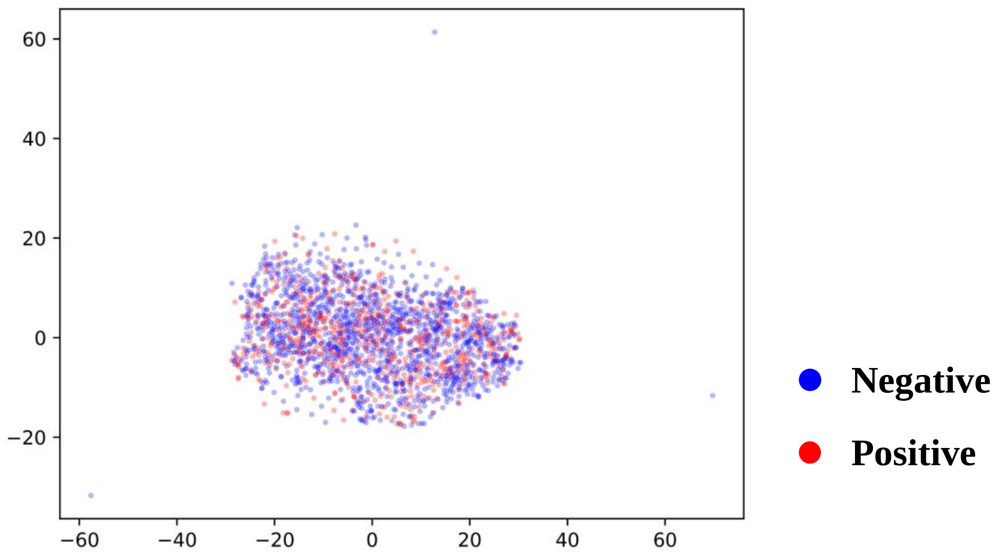

B

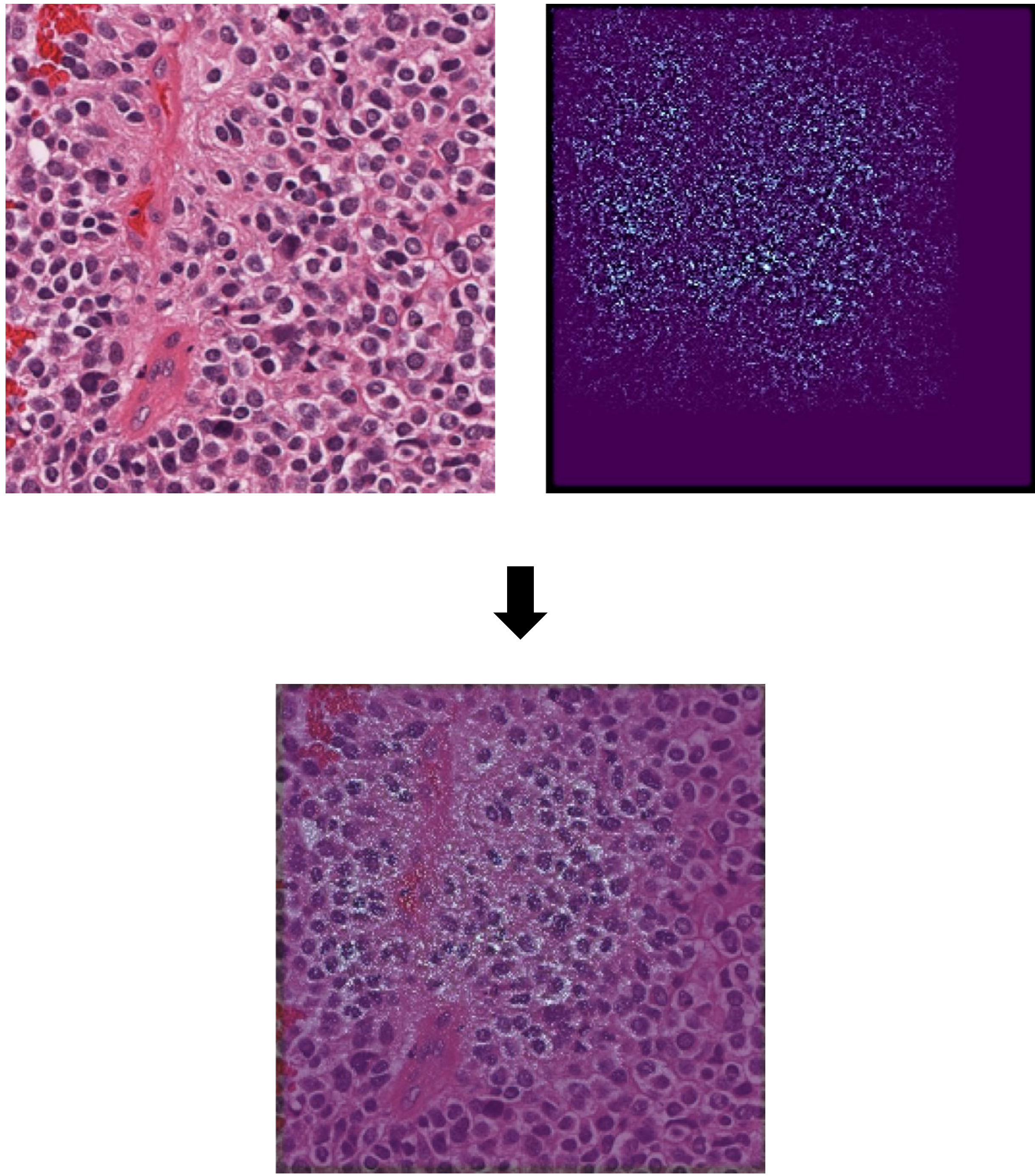

Figure S13. *EGFR* amplification. (A) the t-SNE plot of 1000 random patches (B) An example of pixel-level DeepLift heatmap.

B

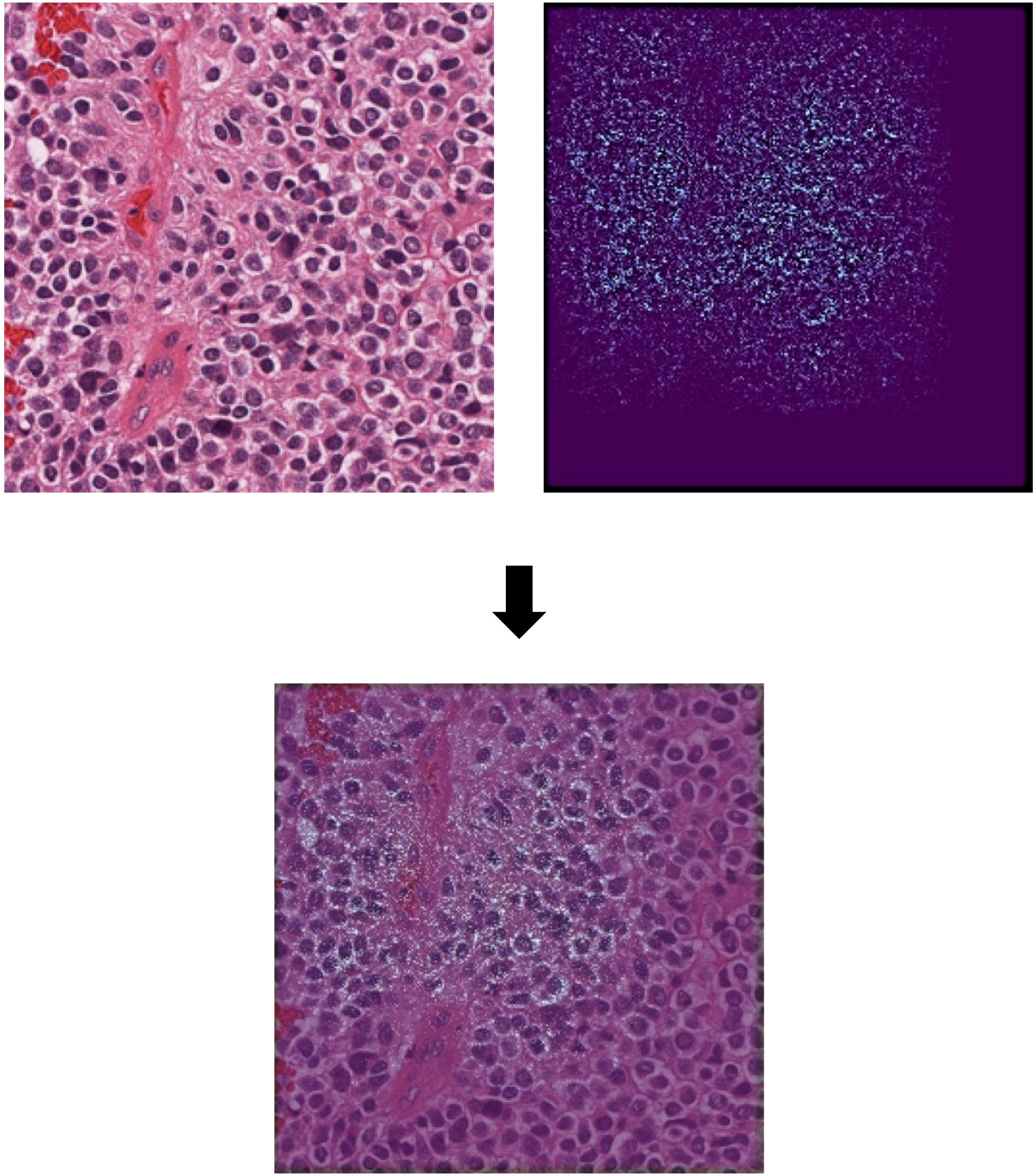

A

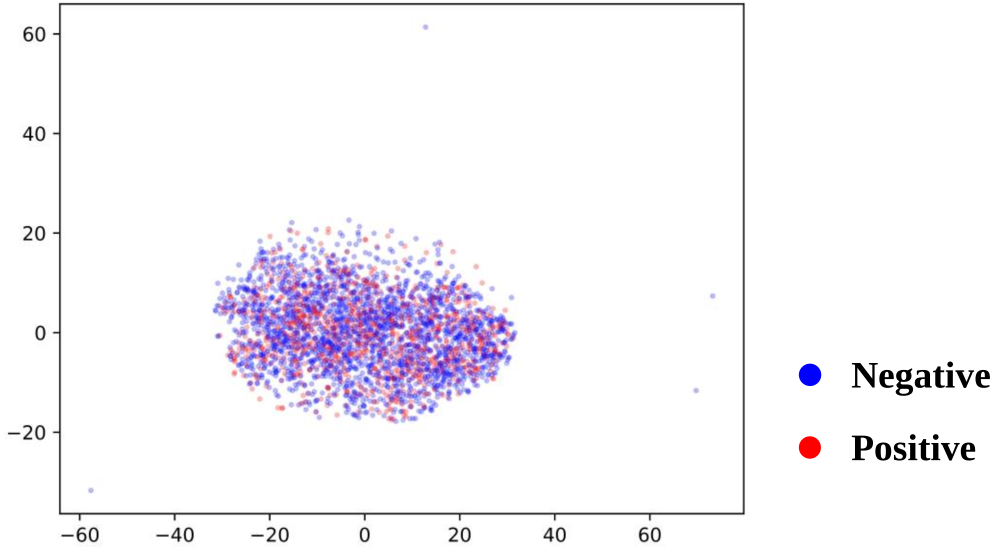

Figure S14. *IDH1/2* mutation. (A) the t-SNE plot of 1000 random patches (B) An example of pixel-level DeepLift heatmap.

A

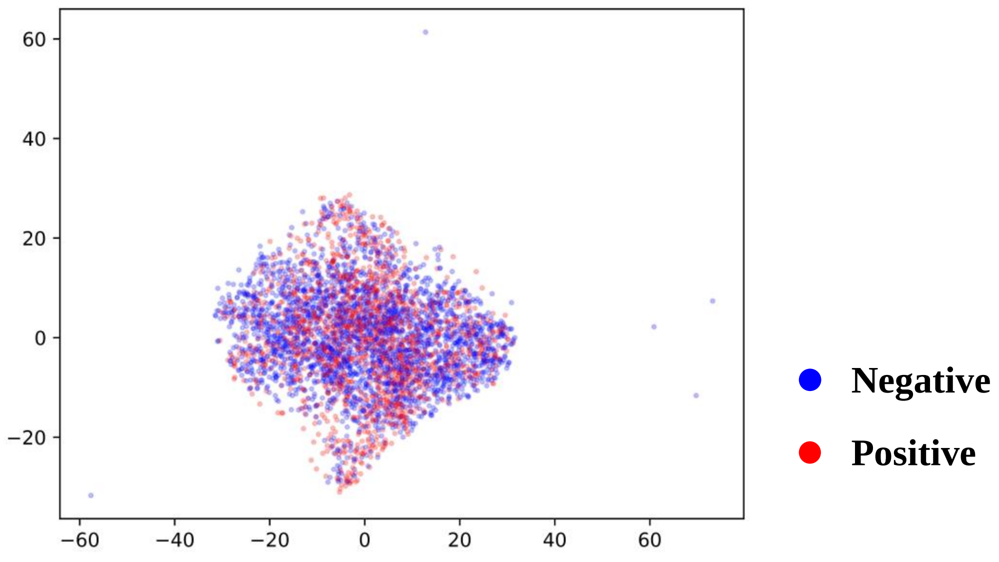

B

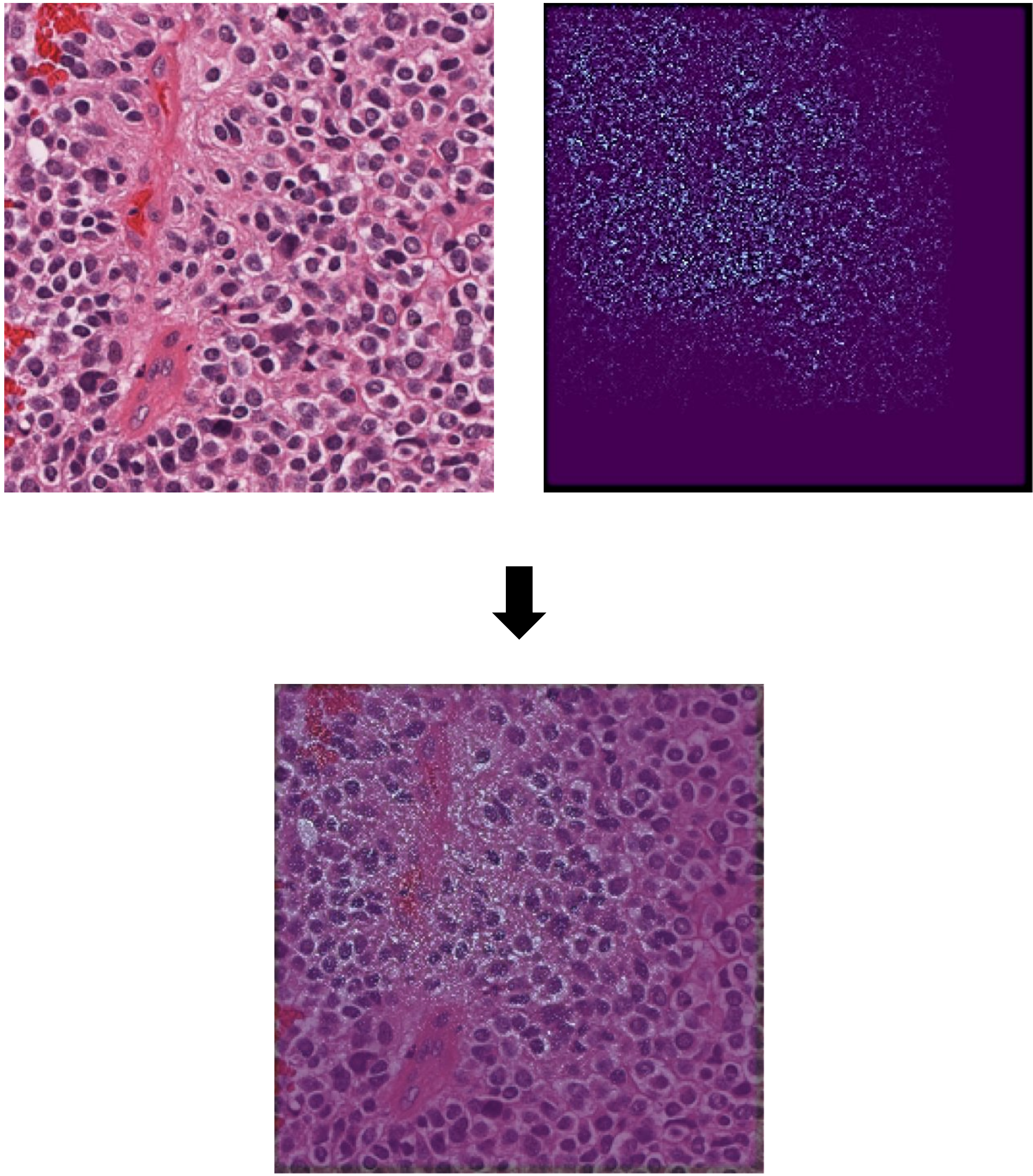

Figure S15. *MGMT* promoter methylation. (A) the t-SNE plot of 1000 random patches (B) An example of pixel-level DeepLift heatmap.

B

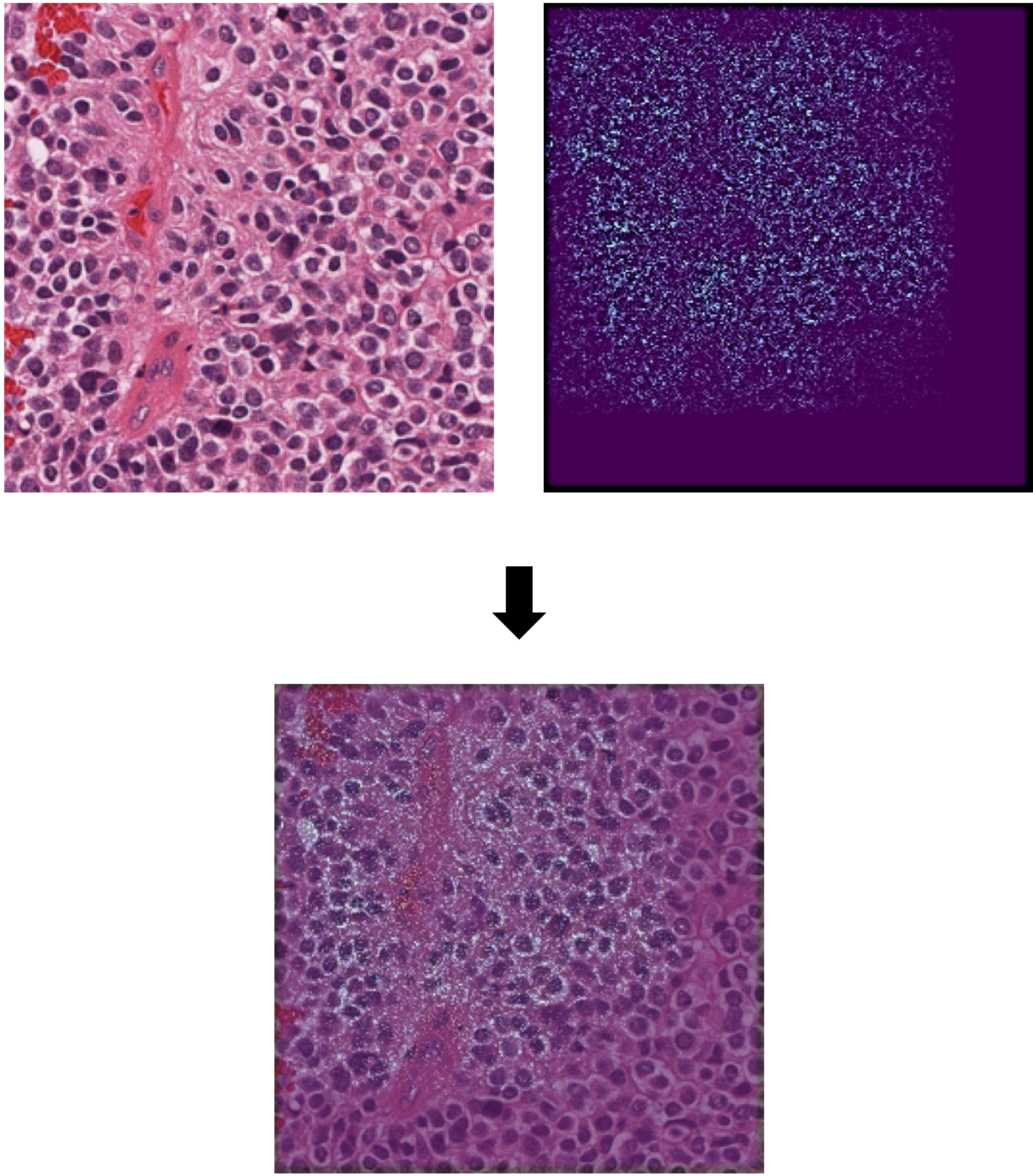

A

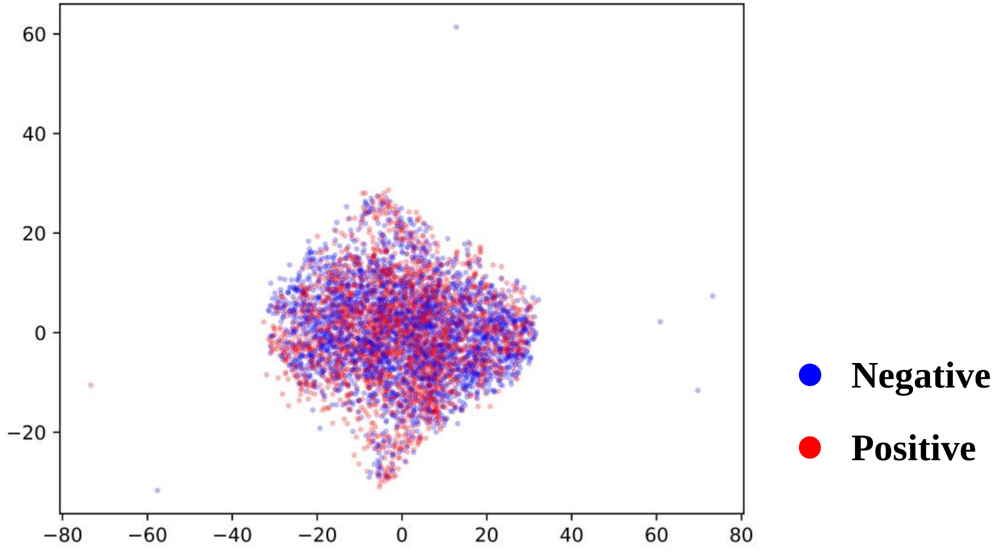

Figure S16. *TERT* promoter mutation. (A) the t-SNE plot of 1000 random patches (B) An example of pixel-level DeepLift heatmap.

B

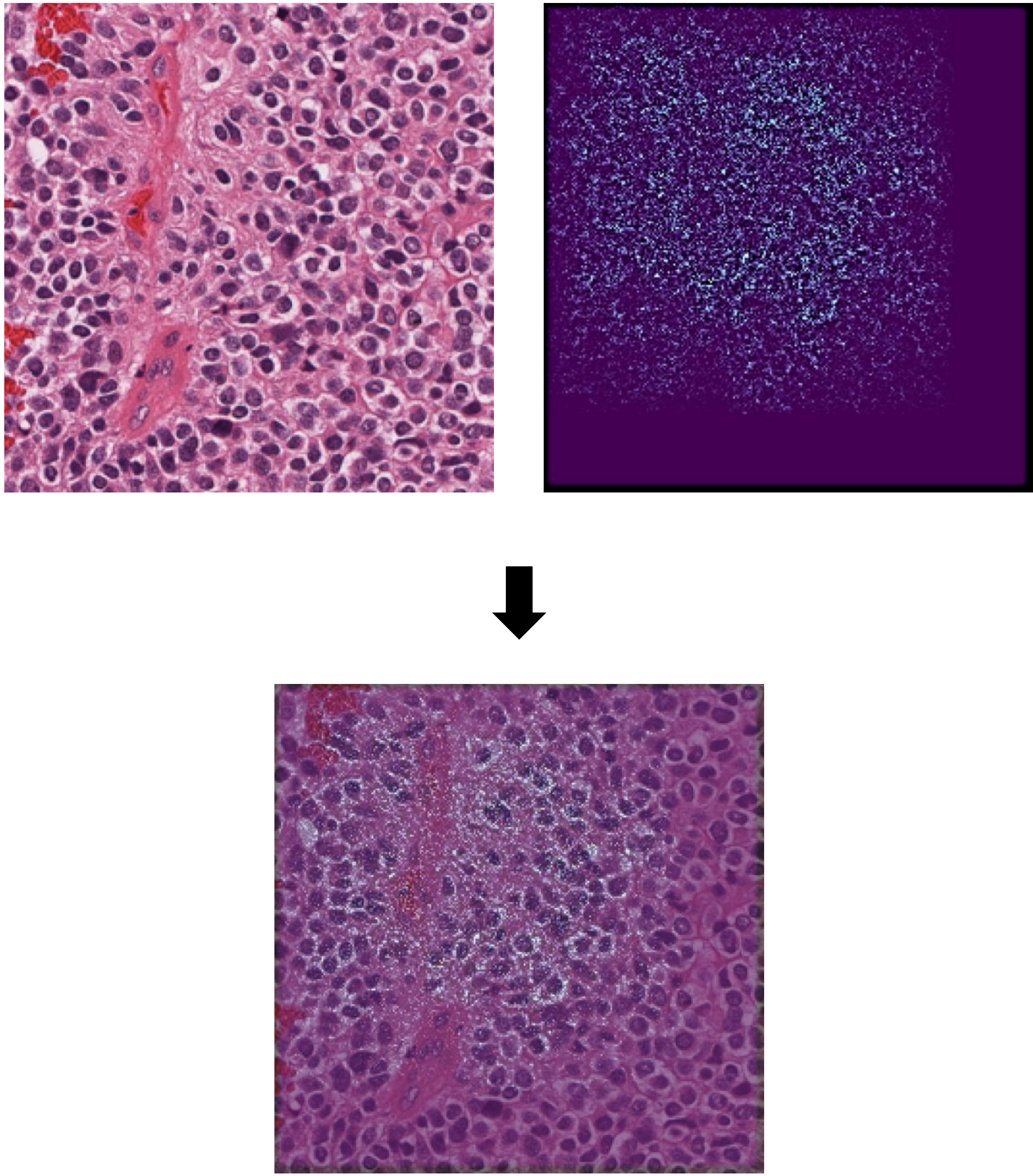

A

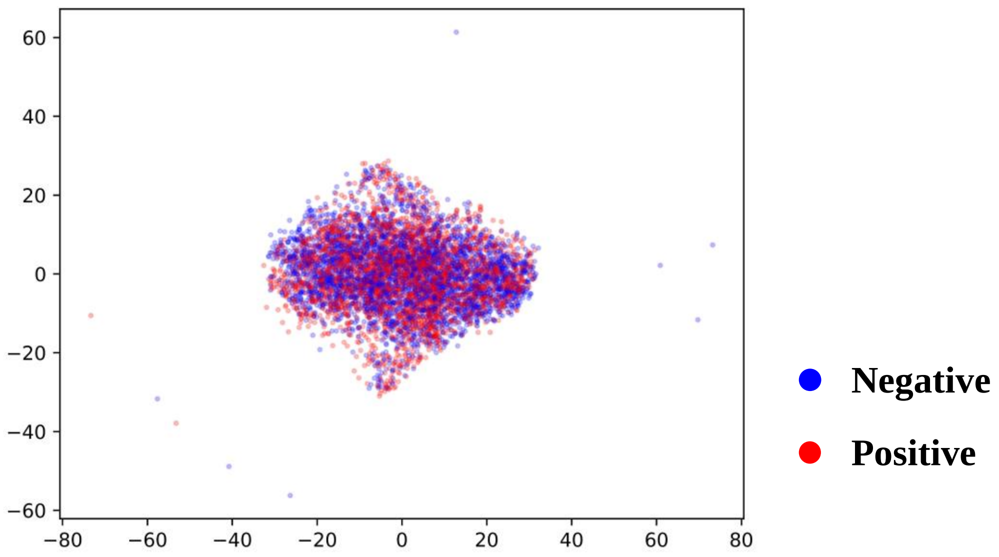

Figure S17. *TP53* mutation. (A) the t-SNE plot of 1000 random patches (B) An example of pixel-level DeepLift heatmap.

B

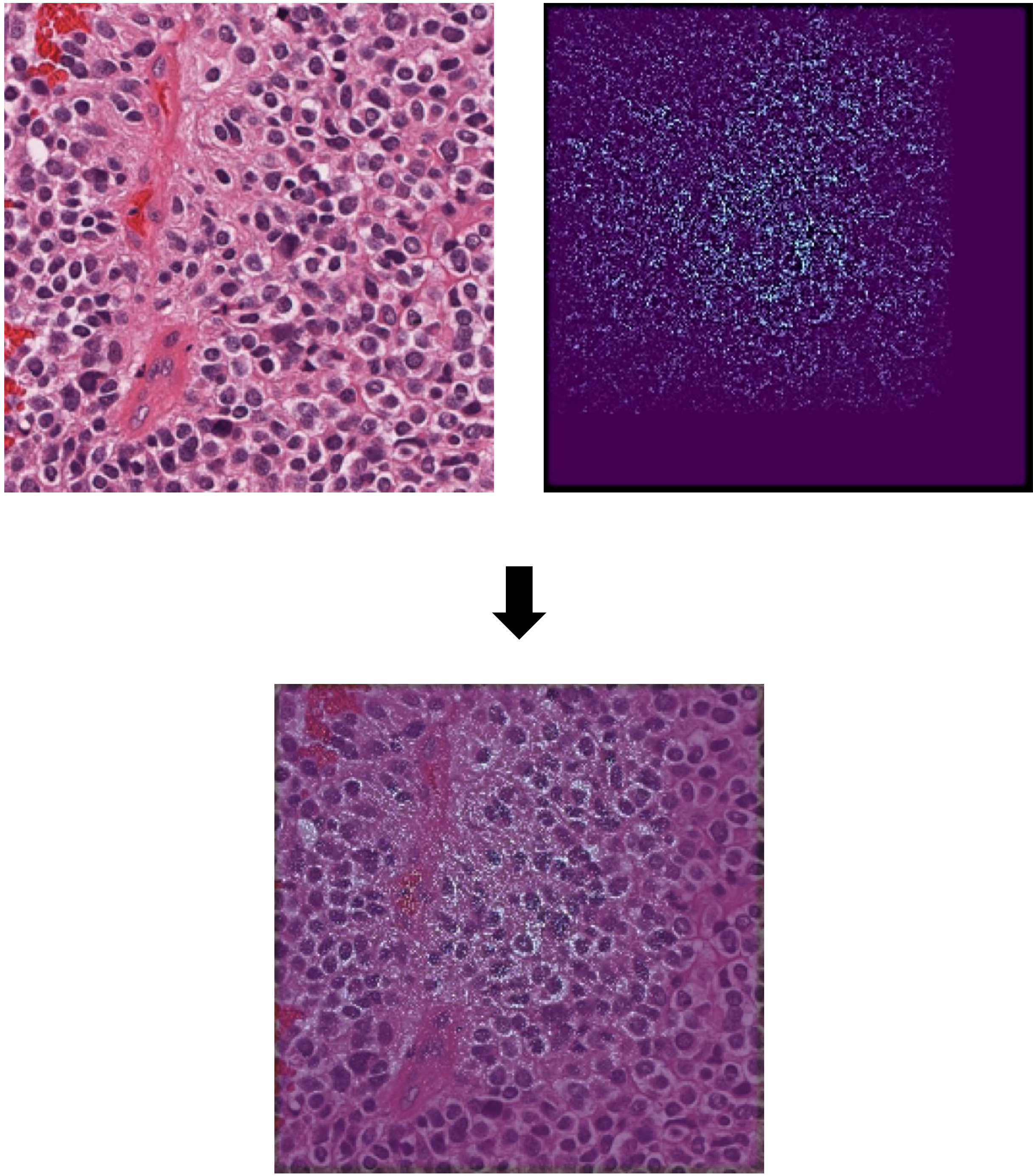

A

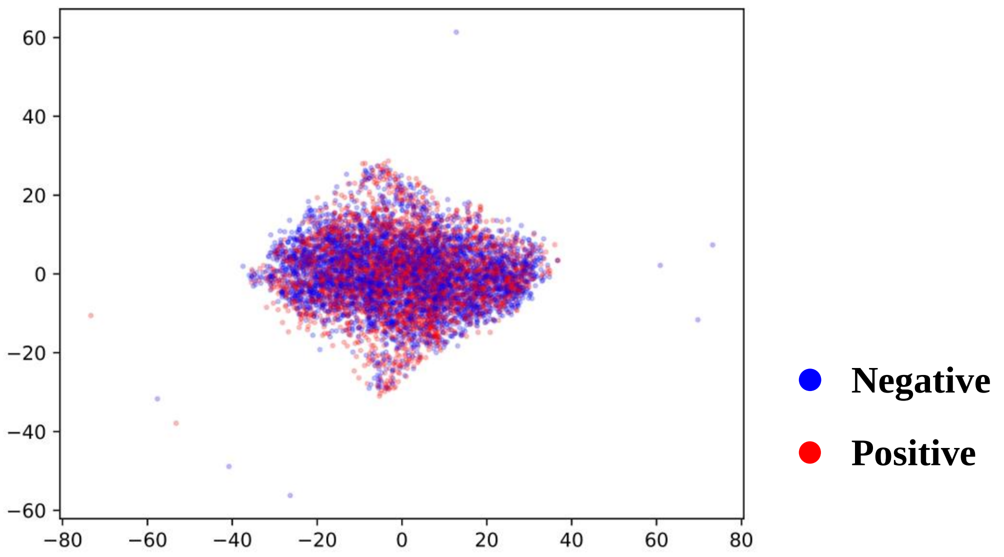

Figure S18. 1p/19q codeletion. (A) the t-SNE plot of 1000 random patches (B) An example of pixel-level DeepLift heatmap.

B

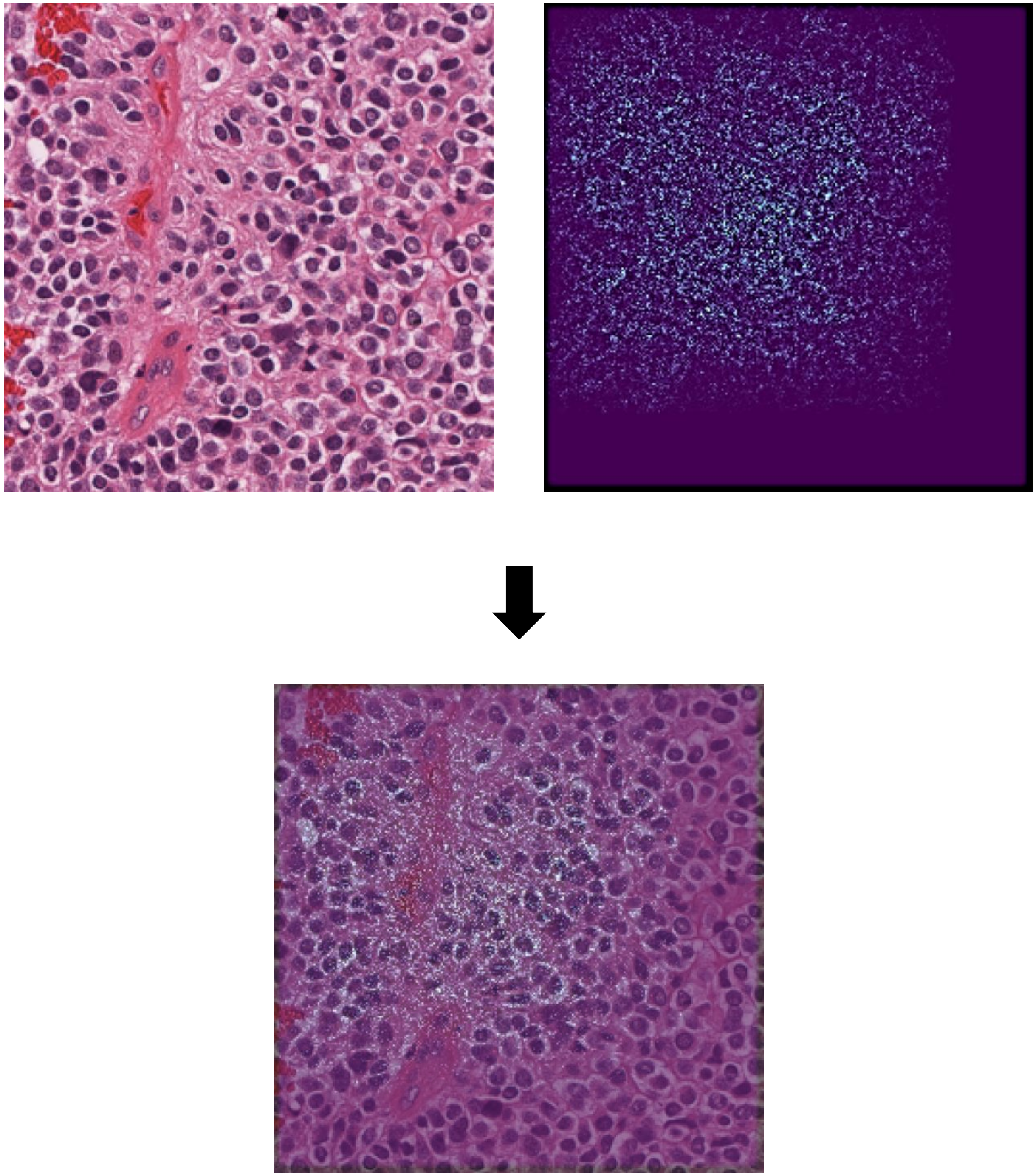

A

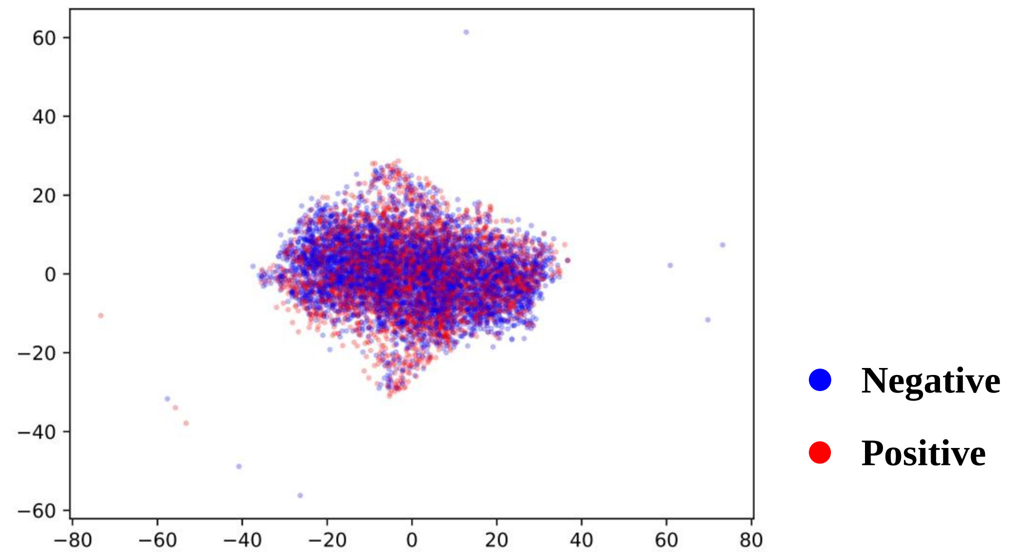

Figure S19. 7 gain/10 loss. (A) the t-SNE plot of 1000 random patches (B) An example of pixel-level DeepLift heatmap.

A

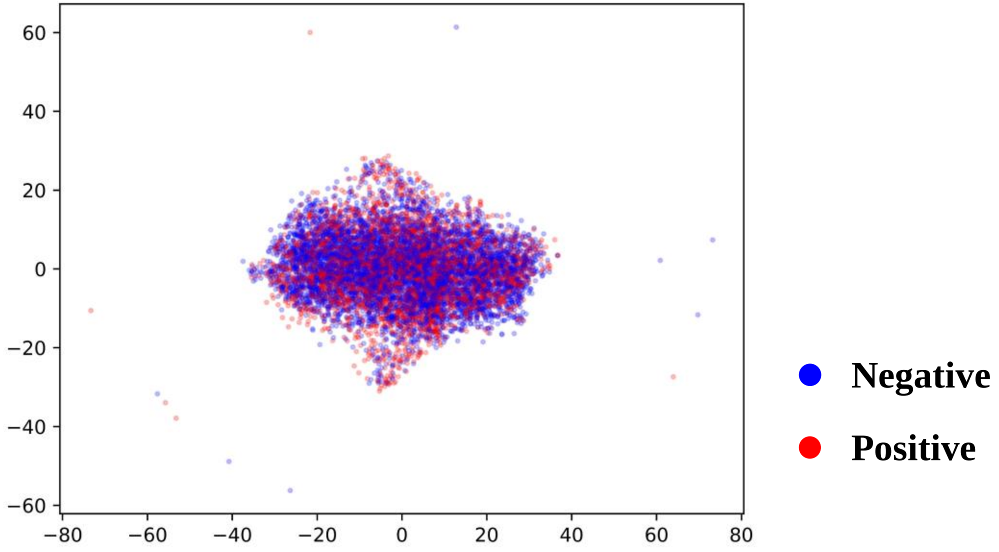

B

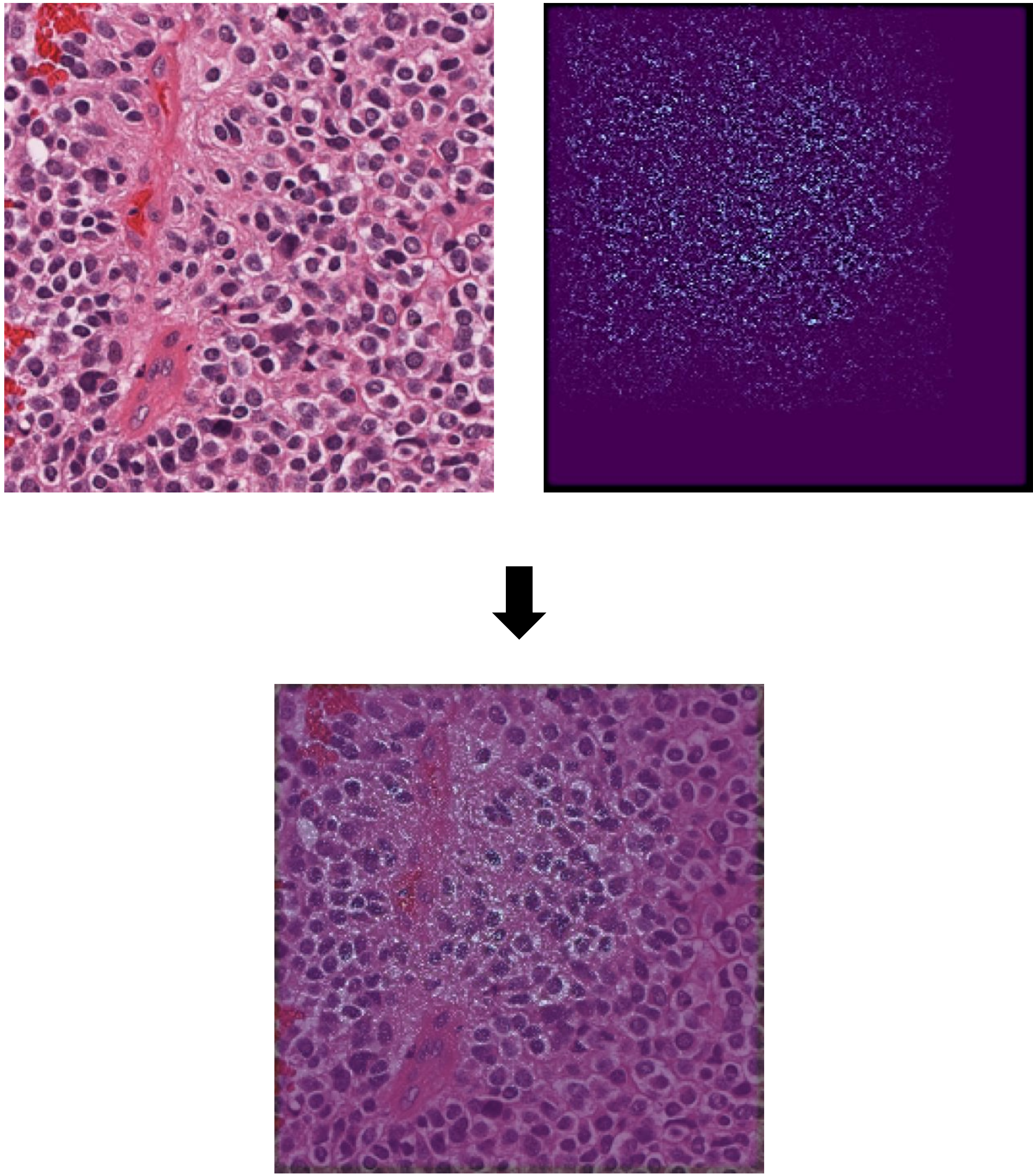

Supplement: Supplementary file 1 [file bioengineering-12-00012-s001.zip › bioengineering-3329217-supplementary.pdf]
